# Proteasome inhibition enhances the anti-leukemic efficacy of chimeric antigen receptor (CAR) expressing NK cells against acute myeloid leukemia

**DOI:** 10.1186/s13045-024-01604-y

**Published:** 2024-09-16

**Authors:** David Sedloev, Qian Chen, Julia M. Unglaub, Nicola Schanda, Yao Hao, Eleni Besiridou, Brigitte Neuber, Anita Schmitt, Simon Raffel, Yi Liu, Maike Janssen, Carsten Müller-Tidow, Michael Schmitt, Tim Sauer

**Affiliations:** Department of Medicine V, Hematology, Oncology and Rheumatology, University Hospital Heidelberg, 69120 Heidelberg, Germany

## Abstract

**Background:**

Relapsed and refractory acute myeloid leukemia (AML) carries a dismal prognosis. CAR T cells have shown limited efficacy in AML, partially due to dysfunctional autologous T cells and the extended time for generation of patient specific CAR T cells. Allogeneic NK cell therapy is a promising alternative, but strategies to enhance efficacy and persistence may be necessary. Proteasome inhibitors (PI) induce changes in the surface proteome which may render malignant cells more vulnerable to NK mediated cytotoxicity. Here, we investigated the potential benefit of combining PIs with CAR-expressing allogeneic NK cells against AML.

**Methods:**

We established the IC50 concentrations for Bortezomib and Carfilzomib against several AML cell lines. Surface expression of class-I HLA molecules and stress-associated proteins upon treatment with proteasome inhibitors was determined by multiparameter flow cytometry. Using functional in vitro assays, we explored the therapeutic synergy between pre-treatment with PIs and the anti-leukemic efficacy of NK cells with or without expression of AML-specific CAR constructs against AML cell lines and primary patient samples. Also, we investigated the tolerability and efficacy of a single PI application strategy followed by (CAR-) NK cell infusion in two different murine xenograft models of AML.

**Results:**

AML cell lines and primary AML patient samples were susceptible to Bortezomib and Carfilzomib mediated cytotoxicity. Conditioned resistance to Azacitidine/Venetoclax did not confer primary resistance to PIs. Treating AML cells with PIs reduced the surface expression of class-I HLA molecules on AML cells in a time-and-dose dependent manner. Stress-associated proteins were upregulated on the transcriptional level and on the cell surface. NK cell mediated killing of AML cells was enhanced in a synergistic manner. PI pre-treatment increased effector-target cell conjugate formation and Interferon-γ secretion, resulting in enhanced NK cell activity against AML cell lines and primary samples in vitro. Expression of CD33- and CD70-specific CARs further improved the antileukemic efficacy. In vivo, Bortezomib pre-treatment followed by CAR-NK cell infusion reduced AML growth, leading to prolonged overall survival.

**Conclusions:**

PIs enhance the anti-leukemic efficacy of CAR-expressing allogeneic NK cells against AML in vitro and in vivo, warranting further exploration of this combinatorial treatment within early phase clinical trials.

**Supplementary Information:**

The online version contains supplementary material available at 10.1186/s13045-024-01604-y.

## Background

The prognosis of patients with acute myeloid leukemia (AML), the most common acute leukemia among adults remains poor despite the approval of several novel drugs during the last two decades. Less than a third of patients are alive 5 years after initial diagnosis [1]. Outcomes are particularly dismal for patients aged 65 years and older [1] and for those with relapsed and/or refractory disease (R/R AML) [2] with a 5-year overall survival (OS) of 10.7% and 12.6%, respectively. This highlights the urgent need for novel therapeutic strategies to improve the outcome of this difficulty-to-treat patient population. The adoptive transfer of autologous CAR T-cells has emerged as a promising treatment strategy for patients with relapsed and refractory hematologic malignancies [3]. However, its application to patients with AML has been challenging for several reasons [4]. First, patient-derived T-cells that are frequently used as source of autologous CAR T-cell products may be functionally impaired, either due to repeated exposure to chemotherapeutic substances during previous treatment episodes or because of the interaction with potentially inhibitory leukemia cells and/or the tumor microenvironment [5]. Additionally, the time-consuming manufacturing process and quality assessments lead to an extended vein-to-vein time (time between collection of T cells and infusion of the CAR-T product) causing problems in clinical implementation [5]. Thus, there is a mounting demand for cellular immunotherapies that can be applied in the allogeneic setting as “off-the-shelf” products. NK cell-based treatment strategies are a promising novel option [6, 7]. NK cells are positioned at the borderline between the innate and adaptive immune system [8] and act as a first line of defense against viral infection and malignant transformation [9]. The adoptive transfer of ex vivo activated and stimulated allogeneic NK cells after haploidentical allotransplant has shown to be feasible and safe [10]. However, further modifications and adjustments are necessary to better exploit the potential of NK cells as therapeutic modality against AML [11]. As an example, NK cells can also be genetically modified to express a CAR and in a pioneering clinical trial, CD19-directed CAR-NK cells have shown to be well-tolerated and effective against B-cell malignancies [12]. The function of NK cells is regulated by the integration of signals coming from activating (e.g. NKG2D, NKp30 and CD16) and inhibitory receptors (KIR protein superfamily) [13]. Activating receptors detect the presence of stress-associated proteins such as the ULBP protein family and MIC-A/B [14], while inhibitory receptors recognize class-I HLA molecules, among others [6, 7]. In AML, a downregulated expression of NKG2D ligands and upregulation of HLA class-I molecules has been shown to induce a shift towards NK cell inhibition [15]. This is compounded by the fact that NK cells isolated from the bone marrow of AML patients show a reduced expression of activating receptors [16]. Thus, enhancing the anti-leukemic efficacy of NK cells against AML cells requires a shift of the signaling balance towards tumor cell recognition and NK cell activation. Preclinical studies have shown that proteasome inhibition modulates the surface proteome of multiple myeloma cells by decreasing the expression of class-I HLA through the inhibition of TAP-dependent peptide-loading [17] while at the same time increasing the expression of stress-associated proteins through the induction of the ER-stress pathway [18].

In the present study, we evaluated the ability of proteasome inhibitors to sensitize AML cells to NK cell mediated killing. We demonstrate that PI pre-treatment induces favorable changes in the AML surface immuno-proteome that enhance the cytotoxicity of peripheral blood derived NK cells in a synergistic manner. We provide evidence that the expression of CARs can further enhance the anti-leukemic potency of activated NK cells against AML. Our data support early phase clinical testing of PI treatment followed by CAR-NK cell therapy for patients with AML.

## Results

### Bortezomib and Carfilzomib exhibit potent cytotoxicity against AML cell lines, primary AML specimens and cell lines conditioned for azacitidine/venetoclax resistance

**Fig. 1:**
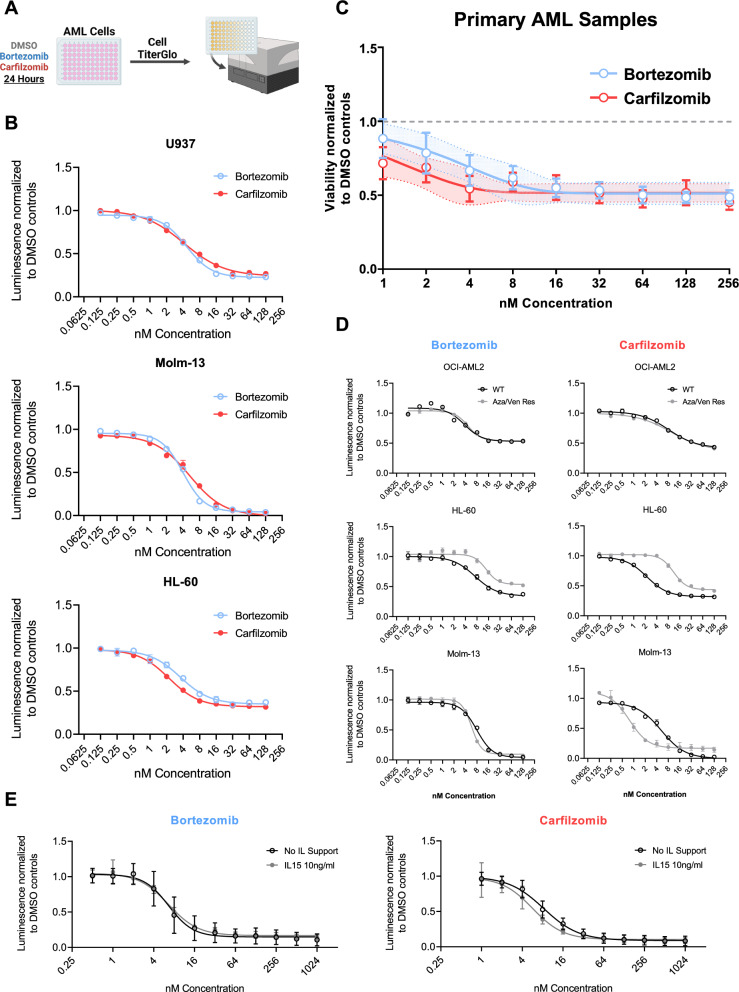
Proteasomal inhibitors effectively inhibit the growth of AML cell lines and primary samples at low nanomolar concentrations. **a** Schematic overview of the Cell-Titer Glo based high-throughput luminescent cytotoxicity assay used to measure the IC50 concentration of Bortezomib and Carfilzomib. Created with BioRender.com **b** IC50 curves following 24-h treatment with Bortezomib (in blue) or Carfilzomib (in red). The luminescent signal at each measured dose level is normalised to the corresponding DMSO controls. Shown are three representative, commonly used AML cell lines—U-937, HL-60 and Molm-13. Values indicated at each dose level are means of three independent experiments ± S.E.M. The IC50 curves of all further cell lines tested can be found in supplementary Figure S1. **c** IC50 curves, compound of six primary samples subjected to high-throughput flow-cytometric analysis after 24 h of exposure to rising doses of Bortezomib (in blue) or Carfilzomib (in red). The viability of each AML sample was normalised to the corresponding DMSO control. Grey interrupted line denotes a normalized viability of 1. All measurements were performed in technical triplicate. Error bars indicate mean ± S.E.M. **d** IC50 curves of three AML cell lines (HL-60, Molm-13, OCI-AML2) with induced resistance to azacitidine and venetoclax (in grey) juxtaposed to wild-type controls of the same cell lines (in black). Values indicated at each dose level are means of three independent experiments ± S.E.M. Statistical comparison of IC50 values between wild-type controls and conditioned cell lines was performed using the Extra-squares of F-test. **e** IC50 curves of activated primary NK cells treated with rising doses of Bortezomib or Carfilzomib for 24 h. The luminescent signal at each measured dose level is normalised to the corresponding DMSO controls. Data represent mean ± S.E.M. of n = 4 biological replicates across two independent experiments

To investigate the susceptibility of AML cells to proteasome inhibitors (PIs) we determined the IC50 concentrations of Bortezomib and Carfilzomib for ten different AML cell lines (HL-60, Molm-13, OCI-AML2 and U937, HEL, IMS-M2, KG1-a, THP1, MV4-11, and K562). The cell lines were exposed to increasing concentrations of Bortezomib and Carfilzomib and cytotoxicity was measured using a high-throughput luminescence-based cell viability assay (Fig. 1A). Multiple myeloma cell lines known to be sensitive to PI treatment [19] (RPMI-8226, Molp-8) served as controls. Nine out of ten wild-type AML cell lines were susceptible to both drugs with a mean IC50 of 5.1 ± 0.6 nM for Bortezomib and 17.2 ± 7.8 nM for Carfilzomib, with the sole exception of K562 cells that were resistant to both compounds (Fig. 1B, Figure S1, S2). Additionally, we tested six primary AML samples and found that treatment with Bortezomib and Carfilzomib led to a relevant decrease in the percentage of viable blast cells, even at concentrations as low as 1 nM (Mean viability normalized to DMSO control: Bortezomib_1nM_ 88.2 ± 13.1%; Bortezomib_2nM_ 78.5 ± 13.8%; Bortezomib_4nM_ 66.8 ± 10.3%; Carfilzomib_1nM_ 71.6 ± 10.8%; Carfilzomib_2nM_ 68.9 ± 10.2%; Carfilzomib_4nM_ 54.4 ± 8.6%) (Fig. 1C). We then explored the impact of PI treatment on three AML cell lines (HL-60, Molm13 and OCI-AML2) that had been conditioned for resistance to treatment with Venetoclax and Azacitidine (VEN/AZA), the current standard therapy for AML patients ineligible for intensive chemotherapy [20]. We found that VEN/AZA-resistant HL-60 cells were less susceptible to PI treatment than wild-type HL-60 (HL-60_WT_ IC50_Bortezomib_ 7.1 nM vs HL-60_Res_ IC50_Bortezomib_ 13.9 nM *P* = 0.0002; HL60_WT_ IC50_Carfilzomib_ 2.2 nM vs HL-60_Res_ IC50_Carfilzomib_ 11.1 nM *P* < 0.0001) while Molm-13_Res_ and were more susceptible than their wild-type controls (Molm-13_WT_ IC50_Bortezomib_ 7.9 nM vs Molm-13_Res_ IC50_Bortezomib_ 5.8 nM *P* < 0.0001; Molm-13_WT_ IC50_Carfilzomib_ 5.3 nM vs Molm-13_Res_ IC50_Carfilzomib_ 0.8 nM *P* < 0.0001). For the OCI-AML2, the VEN/AZA resistant cell line was similar susceptible to proteasome inhibition as the wild type cells (OCI-AML2_WT_ IC50_Bortezomib_ 4.6 nM vs OCI-AML2_Res_ IC50_Bortezomib_ 3.8 nM *P* = 0.2650; OCI-AML2_WT_ IC50_Carfilzomib_ 9.4 nM vs OCI-AML2_Res_ IC50_Carfilzomib_ 8.2 nM *P* = 0.3577) (Fig. 1D, Figure S2).

Next, we determined the impact of PI treatment on the viability of activated NK cells. To this end, we treated healthy donor-derived NK cells (n = 8) 14 days after activation with increasing doses of Bortezomib and Carfilzomib either in the presence or the absence of IL-15, a cytokine frequently used for the support of NK cells ex vivo and in vivo [21]. With a mean IC50 concentration of 6.6 nM for Bortezomib (Range: 5.6–7.9 nM) and 8.3 nM for Carfilzomib (Range: 7.4–9.3 nM) activated NK cells were susceptible to both Bortezomib and Carfilzomib mediated cytotoxicity. Notably, the IC50 concentrations of Bortezomib/Carfilzomib were in a similar range for the tested AML cell lines and activated NK cells. The addition of Interleukin-15 did not affect the susceptibility of activated NK cells to PI (Fig. 1E).

### Proteasome inhibitor pre-treatment downregulates class-I HLAs and induces expression of stress-associated proteins (SAPs) on AML cells in a dose-and-time dependent manner

**Fig. 2:**
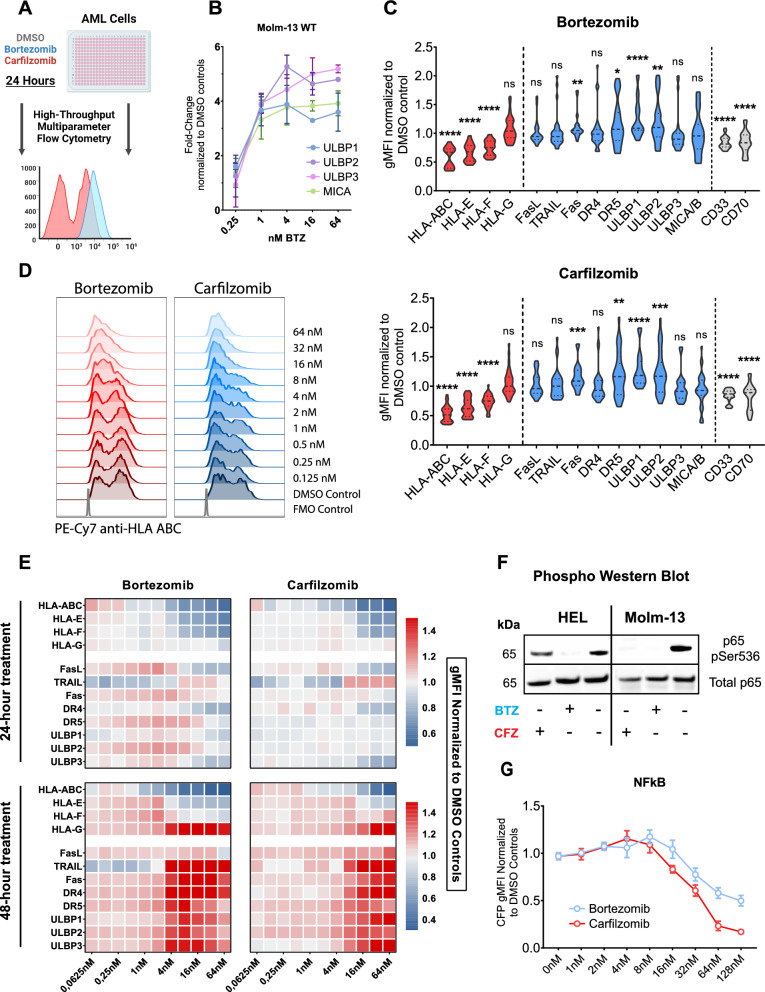
Proteasome inhibition affects a change in the expression levels of class-I HLA molecule and stress-associated proteins. **a** Schematic overview of the flow-cytometric workflow used to measure the impact of proteasome inhibition upon the surface proteome of AML cell lines. Created with BioRender.com **b** RNA was extracted from wild-type Molm-13 cells following a 24-h incubation with rising doses of Bortezomib using a commercially available kit (Qiagen). Data show measurements performed in technical triplicate; error bars indicate mean ± S.E.M. **c** Violin plots of the relative quantitative expression of select proteins on the surface of ten AML cell lines following 24 h of PI treatment with the IC50 concentration. Class-I HLA molecules are represented in red, stress-associated proteins in blue and two AML-associated CAR target proteins in grey. Measurements represent gMFI of each protein on viable cells after normalisation to DMSO-controls. Data shown represent the results of three independent experiments. Statistical comparison by one-sample t-test comparing the DMSO-control normalized ΔgMFI of each measured marker to 1. **d** Representative flow-cytometric histograms of the expression of HLA-ABC on samples from wild-type Molm-13 cells exposed to rising concentrations of Bortezomib (shades of red) or Carfilzomib (shades of blue) in addition to FMO-controls (grey). **e** Heatmap of the relative quantitative expression of select proteins on the surface of wild-type Molm-13 cells under the influence of rising doses of Bortezomib and Carfilzomib. Measurements represent gMFI of each protein on viable cells after normalisation to DMSO-controls. **f** Phospho western blot of p65 and pSer536 p65 in lysates of HEL and Molm-13 cells, two AML cell lines with constitutive NFkB activation. The cells were treated with either a PI at the respective IC50 concentration or a DMSO control for 24 h prior to protein lysate extraction. **g** Jurkat triple-parameter-reporter cells were activated using a commercially available cocktail of PMA and Ionomycin and subjected to rising doses of proteasome inhibitors or DMSO-controls. The gMFI of CFP (NFkB reporter) normalized to DMSO-controls is shown. Measurements are indicative of means across three independent experiments; the error bars indicate ± S.E.M

To determine the effect of PI treatment on the surface expression of class-I HLA molecules and stress-associated proteins, we used a high-throughput multiparameter flow cytometry protocol (Fig. 2A). After 24 h of exposure to Bortezomib and Carfilzomib, we observed a decrease in the expression of the class I HLAs A, B, C, D, E and F but not G for all AML cell lines (Fig. 2C). Surface expression of stress-associated proteins did not increase after 24 h of PI treatment (Fig. 2C). Next, we exposed Molm-13 cells to Bortezomib treatment for 24 h and determined the expression of four different stress-associated protein transcripts (ULBP1/2/3 and MICA/B) by quantitative RT-PCR. PI treatment increased the expression of all four transcripts in a dose-dependent manner (Fig. 2B). Having confirmed stress-associated protein upregulation on the transcriptional level, we extended the treatment period of Molm-13 cells with PIs and found that upregulation of stress-associated protein surface expression was detectable starting 48 h after exposure to PI treatment (Fig. 2D–E). Notably, whereas the intensity of class-I HLA downregulation clearly correlated with PI concentration of the treatment, we observed the highest level of stress-associated protein upregulation at PI doses close to the IC50 concentration for both drugs (Fig. 2E). To compare these results with standard chemotherapeutic agents used to treat AML, we performed the identical process of phenotyping on Molm-13 cells with Daunorubicin and Ara-C (Figure S6). As reported previously, both compounds increase the expression of stress associated proteins in a dose-dependent manner. Neither compound led to a reduction of class-I HLA expression. On the contrary, treatment with Cytarabine led to a strong increase in class-I HLA expression, as recently reported for Bleomycin [22].

An additional proposed mechanism of action of proteasome inhibitors in AML is the reduction of constitutive nuclear factor kappa-light-chain-enhancer of activated B cells (NF-κB) signalling, particularly in leukemic stem cells (LSCs) [23, 24]. To investigate this in detail, we analysed the phosphorylation of p65, one component of the NF-κB transcription factor family, in two AML cell lines with reported constitutive NF-κB activation (HEL, Molm-13) [25] after exposure to PI treatment using a standard Western blot assay. With increasing concentrations of Bortezomib and Carfilzomib we observed a reduction of p65 phosphorylation in cells treated with PI while the total amount of p65 remained unchanged (Fig. 2F). To confirm this result, we utilized a dynamic triple parameter reporter system expressed by Jurkat cells that allows for the simultaneous detection of the activity of the transcription factors NF-κB, NFAT and AP-1 [26]. After activation with PMA and Ionomycin, the Jurkat cells were treated with increasing concentrations of Bortezomib, Carfilzomib or with DMSO as control. We observed a decline in NFkB signaling intensity for both drugs within 12 h of PI-treatment. Exposure to Carfilzomib but not Bortezomib additionally reduced NFAT activity (Fig. 2G; S5A–B).

### Pre-treatment with PIs enhances cytotoxicity of activated NK cells (aNK)

**Fig. 3:**
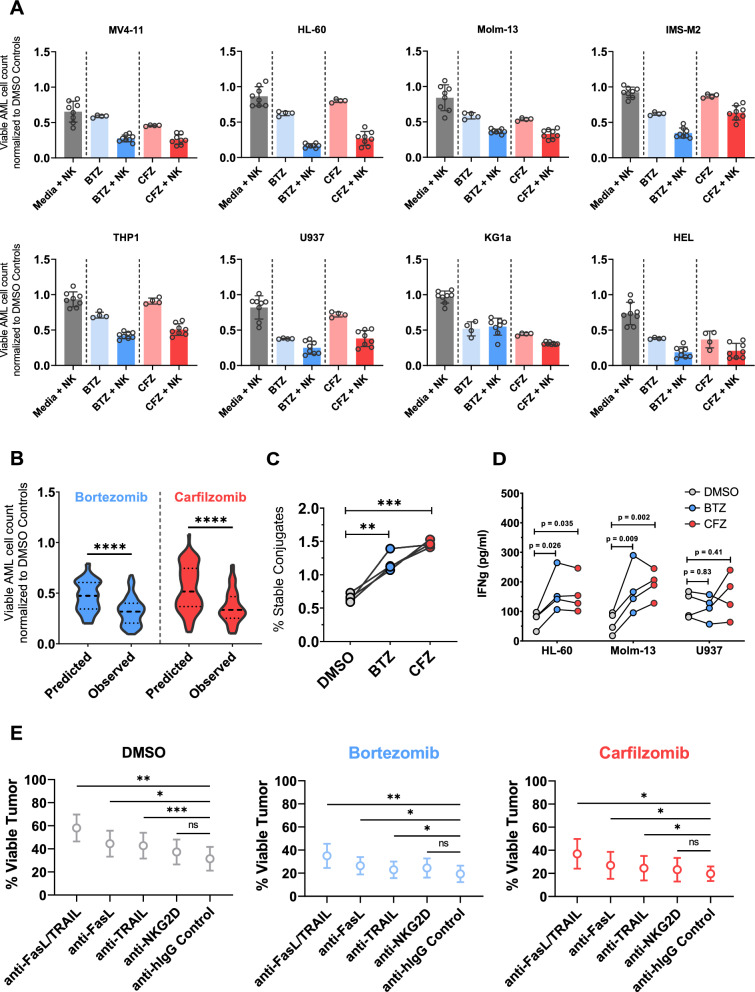
Non-transduced NK cells exhibit potent and synergistic cytotoxicity against a variety of AML cell lines pre-treated with proteasome inhibitors. **a** Surviving AML cell counts after 24 h of co-culture are shown. Data shown are from two independent experiments using n = 8 biological replicates (different PBMC donors). All values are normalized to DMSO-only controls. **b** The predicted combinatorial cytotoxicity was compared to the observed combinatorial cytotoxicity of Bortezomib or Carfilzomib in the eight cell lines shown in 3A. Statistical comparison by unpaired Student t-test. **c** Percentage of stable conjugates formed between aNK and Molm-13 cells pre-treated with DMSO or IC50 Bortezomib (blue) or Carfilzomib (red). N = 4 biological replicates. Statistical comparison by one-way ANOVA followed by post-hoc analysis and Dunnett´s multiple comparison correction **d** Interferon-γ concentration in supernatants from 3A was quantified using a standard ELISA. Statistical comparison by one-way ANOVA and post-hoc Dunnett multiple hypothesis correction. **e** NK-mediated antileukemic cytotoxicity after blocking NKG2D, FasL, TRAIL or FasL and TRAIL simultaneously. Data shown represent means ± S.E.M. of NK cells from three different healthy donors and 11 different AML cell lines. Statistical comparison by one-way ANOVA followed by post-hoc analysis and Dunnett´s multiple comparison correction with the anti-IgG group serving as control *P < .05; **P < .01; ***P < .001, ***P < .0001

To generate activated NK cells (aNKs), we established a robust ex vivo activation and expansion protocol that utilizes CD80, CD83, CD86 and CD37-L expressing K562 derived feeder cells, allowing us to activate and expand peripheral blood NK cells with a median 2200-fold-expansion within 14 days (Figures S3A–B). These aNKs displayed upregulated activating receptors and death ligands as compared to non-stimulated NK cells that are circulating in peripheral blood (gMFI fold-change normalized to NK cell controls isolated from peripheral blood: NKG2D_Day14_: 6.1 ± 0.3; NKG2C_Day14_ 4.5 ± 1; NKp30_Day14_ 3.9 ± 0.2; NKp46_Day14_ 4.9 ± 0.7; FasL_Day14_ 4.4 ± 0.4; TRAIL_Day14_ 5.4 ± 0.2) (Figure S3C-E). To investigate the functional relevance of PI pre-treatment as a sensitizer for NK cell mediated killing, we pre-treated eight different AML cell lines (HL-60, Molm-13, U937, HEL, IMS-M2, KG1-a, THP1, MV4-11) with Bortezomib and Carfilzomib at the previously determined IC50 concentration for 24 h and co-cultured them afterwards with aNK for an additional 24-h in the absence of Bortezomib and Carfilzomib. The combination of PI pre-treatment and aNK cells displayed an enhanced antileukemic cytotoxicity compared to PI pre-treatment or aNK cells alone. To quantify the synergy between the two treatment modalities, we used Jin’s modified Bürgi formula as described previously [27, 28]. Figure 3B shows the predicted and observed cytotoxicity of the combinatorial treatments. Both compounds showed significant synergy with aNK effectors (Combinatorial Index_aNK+Bortezomib_ 1.40 *P* < 0.0001; Combinatorial Index_aNK+Carfilzomib_ 1.728 *P* < 0.0001).

Next, we aimed to explore the underlying mechanism of the enhanced antileukemic potency of the combinatorial treatment by investigating the effects of PI pre-treatment on the degranulation-dependent NK cytotoxicity pathway and the degranulation-independent, death ligand induced apoptosis pathway in AML. To this end, we incubated aNKs with PI- or DMSO-pre-treated Molm-13 cells and measured the formation of stable conjugates between aNKs and Molm-13 cells (Figure S9). The percentage of stable conjugates was higher after pre-treatment with PIs compared to DMSO (Stable conjugate formation promille: Molm-13_DMSO_ 6.5 ± 0.3‰ vs Molm-13_Bortezomib_ 11.8 ± 0.7‰ vs Molm-13_Carfilzomib_ 14.6 ± 0.2‰) (Fig. 3C). Furthermore, we found that PI pre-treatment of HL60 and Molm-13 but not U937 cells significantly increased secretion of interferon-γ (IFNγ) by aNKs compared to the DMSO controls (IFNγ concentration in pg/ml for HL-60_DMSO_ 73.4 ± 14.3 vs HL-60_Bortezomib_ 165.9 ± 34.3 vs HL-60_Carfilzomib_ 157.2 ± 31.6; Molm-13_DMSO_ 61.5 ± 18.1 vs Molm-13_Bortezomib_ 173.7 ± 41.3 vs Molm-13_Carfilzomib_ 192.2 ± 24.4; U-937_DMSO_ 121.5 ± 22.3 vs U-937_Bortezomib_ 113.3 ± 21 vs U-937_Carfilzomib_ 152.8 ± 38.1) (Fig. 3D).

Preclinical data using multiple myeloma cells points to death ligand induced apoptosis as the main driver of NK mediated cytotoxicity against PI-treated cells [29]. In contrast, AML cells have been shown to be resistant to death ligand induced apoptosis due to their low baseline expression level of death receptors, as well as their ability to secrete decoy death receptors and block death ligands [30]. The aNKs expressed more death ligands on their cell surface compared to circulating peripheral blood NK cells (Figure S3C-E), while PI treatment led to increases in the surface expression of Fas and DR4 on AML cells (Fig. 2B). We sought to determine the impact of PI pre-treatment on the sensitivity of AML cells to death-ligand induced apoptosis. We pre-treated ten different AML cell lines with IC50 doses of Bortezomib, Carfilzomib, or a DMSO control for a duration of 24 h and incubated them for 24 h with 100 ng/ml of FasL, 100 ng/ml of TRAIL or a PBS control after PI removal (Figure S10A). Pre-treatment with Bortezomib or Carfilzomib led to substantially lower AML viability and enhanced the cytotoxicity of FasL and TRAIL (Figure S10B-C). The combination between soluble death ligands and PI pre-treatment was synergistic (Figure S10 D-E).

Using Molm-13 cells, we show that blockade of class-I HLA molecules leads to enhanced NK mediated killing (Figure S11C), as reported previously for melanoma [31]. However, blocking NKG2D did not reduce the killing of Molm-13 cells (Figure S11C). Speculating that the failure of NKG2D blocking to decrease killing was a consequence of the low NKG2D ligand expression on Molm-13 (Figure S11D), we performed a series of blocking experiments with neutralizing monoclonal antibodies using eleven different AML cell lines. We show that the inhibition of FasL or TRAIL significantly reduces NK-mediated killing of tumor cells (Fig. 3E). Blocking both FasL and TRAIL further decreases antileukemic efficacy. Blocking NKG2D leads to a reduction in AML cell death, but the effect was not significant (Fig. 3E).

Collectively, these data demonstrate that the pre-treatment of AML cells with PIs synergizes with the antileukemic activity of aNKs.

### Single-dose Bortezomib pre-treatment combined with non-transduced NK cells induces a potent anti-tumor response against AML in vivo

**Fig. 4:**
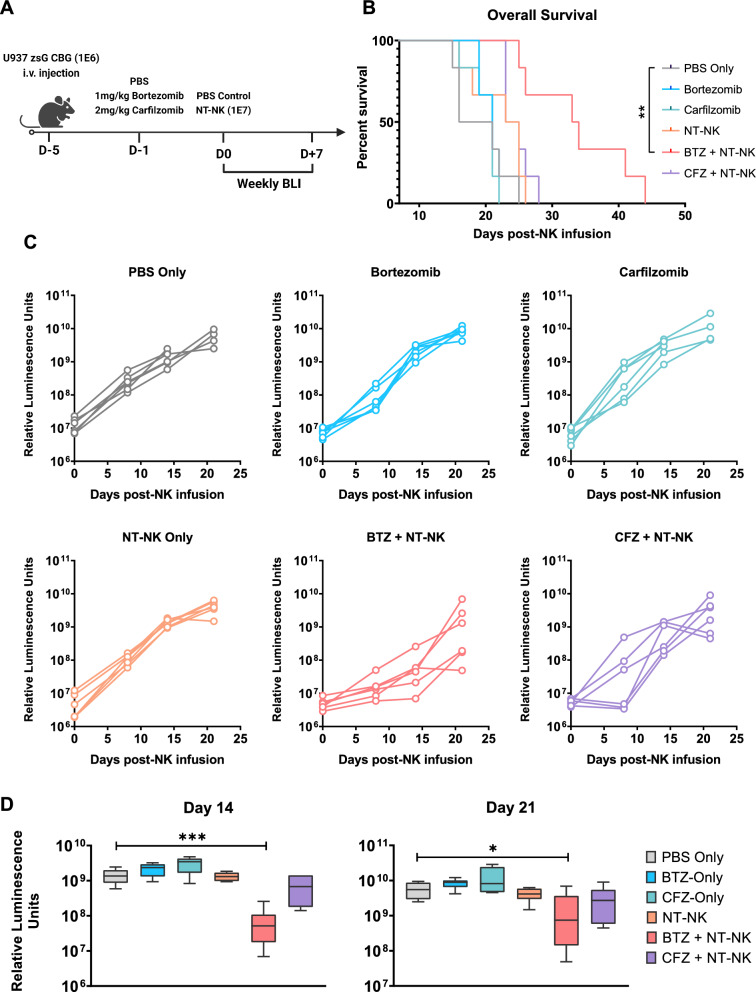
Single-dose Bortezomib pre-treatment leads to significant overall survival improvements in an aggressive AML xenograft model. **a** Short summary of the experimental setup. On day -5, AML engraftment was initiated through the intravenous injection of 1E6 U-937 cells. Proteasome inhibitors (Bortezomib, Carfilzomib or a vehicle-only control) at the stated quantities were injected intravenously four days later. Non-transduced, feeder-cell activated NK cells (1E7 in 200 μl PBS) or a PBS-only control were injected intravenously 24 h after the single-dose proteasome inhibitor treatment. BLI was performed immediately prior to NK cell injections and repeated weekly thereafter (n = 6 animals in each treatment group). Created with BioRender.com **b** Kaplan–Meier survival plot of the animals in the groups outlined above. The log-rank (Mantel-Cox) test was used when performing the statistical analyses of survival differences between the experimental groups **c** Quantitative analysis of the luminescent intensity of each group throughout the course of the experiment. The time course of each individual animal has been plotted separately. **d** Quantitative comparison of luminescent signals between each group as measured on days 14 and 21 post NK cell infusion. Results are represented as means ± S.E.M. *P < .05; **P < .01; ***P < .001, ***P < .0001 by unpaired Student t test

Our data established synergism between PI pre-treatment and NK cell-mediated cytotoxicity in vitro. We analysed these effects in vivo using a murine xenograft model of aggressive AML. For this purpose, NSG mice were engrafted with 1 × 10^6^ U-937 cells that were genetically modified to express zsGreen and click beetle green (CBG) luciferase (U-937.zsG.CBG). Four days later, the animals were treated with either Bortezomib, Carfilzomib or a vehicle-only control. After an additional 24 h, all mice were infused with 1 × 10^7^ activated NK cells. Tumor growth was followed by weekly bioluminescence imaging (BLI) (Fig. 4A). Neither PI pre-treatment nor the administration of aNKs alone or in combination with Carfilzomib could control tumor growth compared to the vehicle-only control treatment (Luminescence intensity on Day 14: U-937_Untreated_ 14.3 ± 2.8E8 p/s vs U-937_Bortezomib_ 21.9 ± 2.8E8 p/s vs U-937_Carfilzomib_ 30.9 ± 6.1E8 p/s vs U-937_aNK_ 13.4 ± 1.5E8 p/s vs U-937_Carfilzomib+aNK_ 7.6 ± 2.6E8 p/s) (Fig. 4C, Figure S12A-B). In contrast, pre-treatment with Bortezomib followed by aNK cell infusion delayed AML progression (Luminescence intensity on Day 14 U-937_Bortezomib+aNK_ 0.8 ± 0.4E8 p/s; *P* = 0.0008) (Fig. 4C–D) and survival of mice treated with Bortezomib followed by aNK cells was prolonged (Median survival U-937_Untreated_ 18,5 days vs U-937_Bortezomib_ 21 days vs U-937_Carfilzomib_ 21 days vs U-937_aNK_ 24 days vs U-937_Bortezomib+aNK_ 33,5 days vs U-937_Carfilzomib+aNK_ 24 days; *P* = 0.001) (Fig. 4B). Both the Bortezomib and the Carfilzomib combination treatments were well tolerated as none of the animals experienced a significant weight loss, defined as a bodyweight loss of ≥ 10% compared to the weight measured immediately preceding PI- or NK cell treatment, in any of the treatment arms (Figure S12C).

### CAR expression further enhances the antileukemic efficacy of NK cells against PI-treated AML cells

**Fig. 5:**
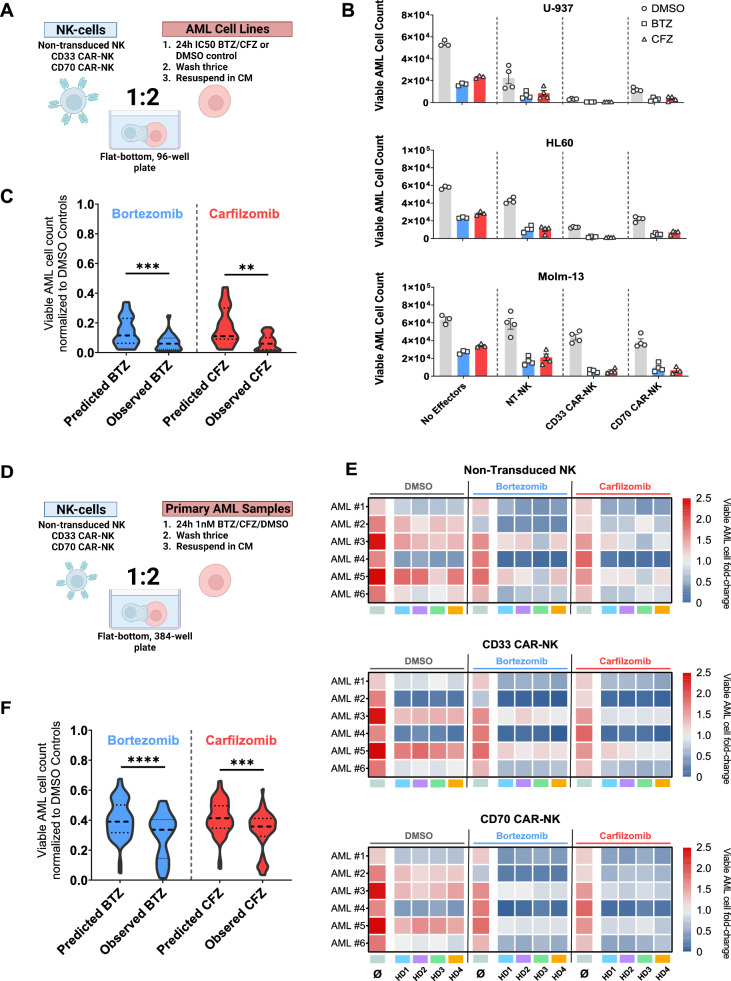
CAR-NK cells outperform non-transduced; feeder cell activated controls and further improve synergistic antileukemic efficacy after PI pre-treatment. **a** Summary of the experimental setup. Following 24-h pre-treatment with PI, AML cells (HL-60, Molm-13 and U-937) were tagged with CellTracker Green and dispensed in 96-well plates as described in Fig. 3A. Created with BioRender.com **b** Histograms showing the antileukemic efficacy of aNK, CD33 CAR-NK and CD70 CAR-NK with and without PI pre-treatment. DMSO-control pre-treatment is shown in grey, Bortezomib in blue and Carfilzomib in red. N = 4 biological replicates. All effector-free incubations were performed in technical triplicate. **c** The predicted combinatorial cytotoxicity was compared to the observed combinatorial cytotoxicity of Bortezomib or Carfilzomib in the three cell lines shown in Fig. 5A. Statistical comparison by unpaired Student t-test. **d** Setup of primary AML sample cytotoxicity screening experiment. As in Fig. 3A and Fig. 4A, primary AML cells derived from six different patients were exposed to PI pre-treatment, then tagged using CellTracker Green and co-cultured with allogeneic NK cells for 24 h. **e** Heatmap of viable AML fold-change normalised to the seeding counts at the beginning of the experiment. **f** Predicted and observed cytotoxicity of the combinatorial treatment with CAR-NK and PIs against primary AML cells. Statistical comparison by unpaired Student t-test. *P < .05; **P < .01; ***P < .001, ***P < .0001

We analysed whether expression of an AML directed CAR construct in NK cells could further enhance their antileukemic potency. We generated CAR-NK cells with two different specificities (Figure S13A)—an scFv-based third-generation CAR targeting CD33 (CD33CAR) and a ligand-based second-generation CAR targeting CD70 (CD70CAR) (Figure S13B). We previously demonstrated preclinical antileukemic activity for both CAR constructs using a T-cell platform [32, 33]. We achieved a high rate of stable transduction with a median transduction efficiency of 94% (Range: 91–96%) and 76% (Range: 72–79%) for the CD33 CAR-NK and CD70 CAR-NK, respectively (Figure S13C). To assess their immediate cytotoxicity, we incubated both CAR-NK cell products with HL-60, U-937 and Molm-13 cells either with or without prior PI treatment (Fig. 5A). Expression of a CD33 or a CD70 specific CAR construct improved the short-term killing capacity of transduced NK cells. Efficacy was confirmed in three target cell lines compared to non-CAR expressing aNK controls (Viable cells normalized to the respective DMSO control: Molm-13_aNK_ 92.3 ± 9.6% vs Molm-13_CD33 CAR-NK_ 68.8 ± 5% vs Molm-13_CD70 CAR-NK_ 60.5 ± 5.3%; Viable U-937_aNK_ 41.8 ± 9.6% vs U-937_CD33 CAR-NK_ 6 ± 0.4% vs U-937_CD70 CAR-NK_ 21.8 ± 1.5%; Viable HL-60_aNK_ 74 ± 2.6% vs HL-60_CD33 CAR-NK_ 21.8 ± 6.3% vs HL-60_CD70 CAR-NK_ 38 ± 2.5%) (Fig. 5B–C). While PI pre-treatment led to a moderate loss of target antigen expression (CD33 ΔMFI_Bortezomib_ 15.4 ± 2.5% and ΔMFI_Carfilzomib_ 21.2 ± 3.1%; CD70 ΔMFI_Bortezomib_ 32.1 ± 4.9% and ΔMFI_Carfilzomib_ 43.9 ± 2.8%) (Fig. 2C), the observed synergy for the combinatorial treatment of PI and CAR-NK cells was similar to PI and aNKs (Combinatorial Index_CAR-NK+Bortezomib_ 1.193 *P* < 0.0006; Combinatorial Index_CAR-NK+Carfilzomib_ 1.243 *P* < 0.007). Figure 5C shows the predicted and observed combinatorial cytotoxicity of CAR-NK cells and PIs. We analysed the anti-leukemic efficacy of CAR-NK cells against six primary AML patient samples with CD33 and CD70 expression (Figure S14A-B) either with or without prior PI treatment (Fig. 5D). Overall, primary patient samples of AML were less susceptible to both aNK- and CAR-NK-mediated killing than most AML cell lines in a 24-h co-culture assay (Viable AML cells normalized to the respective DMSO controls: pAML_aNK_ 56.8 ± 3.4% vs pAML_CD33 CAR-NK_ 46.4 ± 5.1% vs pAML_CD70 CAR-NK_ 54.4 ± 3%) (Fig. 5F, Figure S14C). However, pre-treatment with PIs enhanced the efficacy of both aNKs and CAR-NKs against primary AML samples in a synergistic manner (Combinatorial Index_CAR-NK+Bortezomib_ 1.285 *P* < 0.0002; Combinatorial Index_CAR-NK+Carfilzomib_ 1.131 *P* < 0.0069). The predicted and observed combinatorial cytotoxicity against primary AML cells is shown in Fig. 5F. Additionally, we explored the antileukemic activity of PI treatment in combination with CAR-NK cells against AZA/VEN resistant AML cell lines (Figure S15). In general, these cells were less susceptible to NK-mediated killing than their wild-type counterparts (Figure S15B-C). CD33 and CD70 directed CAR-NKs killed resistant HL-60 cells (HL-60_Res_) but not the AZA/VEN resistant Molm-13 cells (Molm-13_Res_) cell line (Figure S15D). Pre-treating these resistant cell lines with proteasome inhibitors significantly increased the ability of NK cells to kill them (Figure S15E).

We set up a concurrent treatment model in vitro by performing serial re-challenge assays in the presence of proteasome inhibitors (Figure S16). In these assays, we observed an initial increase in antileukemic efficiency at the time point of the first measurement, with lower viable AML cell counts as compared to the DMSO controls. However, the improvement of antileukemic activity was accompanied by a rapid decrease in NK cell viability due to proteasome inhibitor induced toxicity (Figure S16C, S17). As a result, at the second measurement time point, the co-treatment groups performed worse than the DMSO controls (Figure S17C).

In conclusion, the integration of an AML-directed CAR construct improved the performance of aNK cells against antigen positive AML cell lines but was not sufficient to enhance the killing of primary AML samples or AZA/VEN resistant cell lines. PI pre-treatment sensitized these resistant targets to NK mediated killing.

### Synergism between *CAR*-NK cells and PI pre-treatment leads to superior anti-leukemic activity in murine xenograft models

**Fig. 6:**
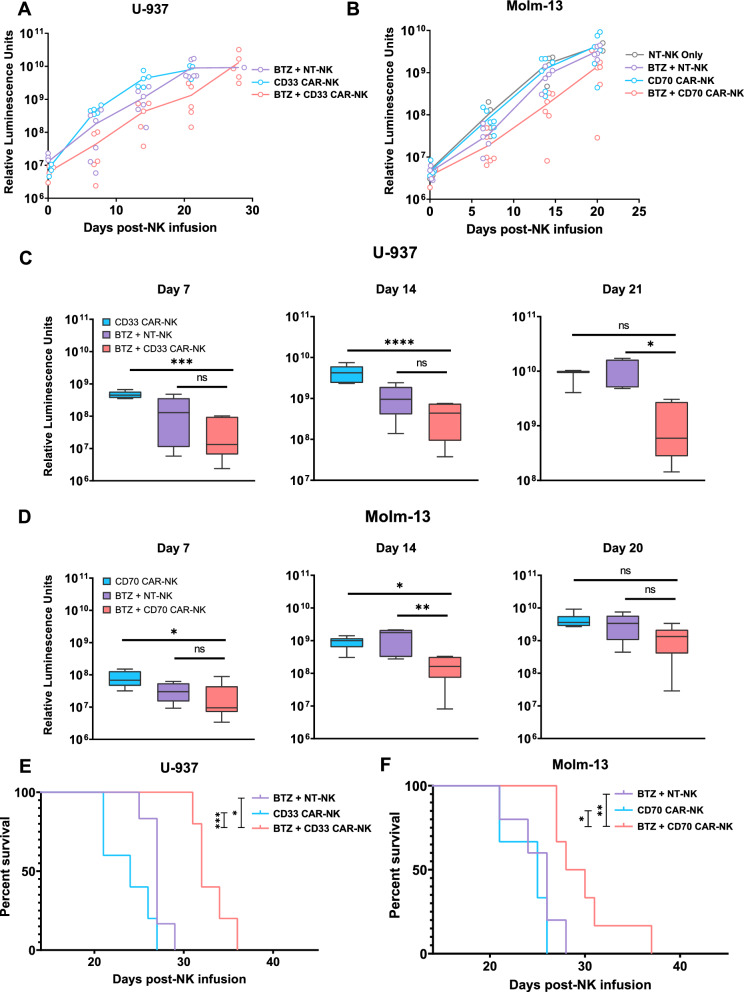
CD33 and CD70 CAR-NK show improved overall survival and leukemic control as compared to non-transduced, activated NK cells **a**, **b** Quantitative analysis of the luminescent intensity of each group throughout the course of the experiment. The time course of each individual animal has been plotted separately at each time point, the line signifies the mean luminescence intensity value of each group. Figure 6C shows the development of luminescence intensity in animals of the U-937 model, Fig. 6D that of the Molm-13 model. **c**, **d** Quantitative comparison of luminescent signals between each group as measured on days 7, 14 and 21 after the day of NK cell injection. **e** Kaplan–Meier survival plot of the U-937 AML model. The log-rank (Mantel-Cox) test was used when performing statistical analyses of survival differences between the experimental groups **f** Kaplan–Meier survival plot of the Molm-13 AML model. The log-rank (Mantel-Cox) test was used when performing statistical analyses of survival differences between the experimental groups. *P < .05; **P < .01; ***P < .001, ***P < .0001 by unpaired Student t test

We analysed whether PI pre-treatment in combination with CD33 or CD70 CAR-NK infusion could enhance anti-leukemic efficacy in vivo. The experimental set-ups are shown in Figures S19A and S20A. In both models, the combinatorial therapy of Bortezomib pre-treatment and subsequent CAR-NK infusion outperformed a single CAR-NK cell infusion (Fig. 6A–D). This effect was associated with prolonged overall survival (Median survival U-937_CD33 CAR-NK_ 24 days vs U-937_Bortezomib+CD33 CAR-NK_ 32 days; *P* = 0.0017; Molm-13_CD70 CAR-NK_ 25 days vs Molm-13_Bortezomib+CD70 CAR-NK_ 29 days; *P* = 0.0007) (Fig. 6E–F). Comparing aNK to CAR-NK as combinatorial partners to PI pre-treatment, CAR-NK cells achieved a lower tumor burden at each measurement point, but only crossed the threshold of significance on day 21 in the U-937 model and day 14 in the Molm-13 model, respectively (Fig. 6E–F). Both models showed superior survival for the CAR-NK + PI group as compared to the aNK + PI control (Median survival U-937_Bortezomib+aNK_ 27 days vs U-937_Bortezomib+CD33 CAR-NK_ 32 days; *P* = 0.0014; Molm-13_Bortezomib+aNK_ 26 days vs Molm-13_Bortezomib+CD70 CAR-NK_ 29 days; *P* = 0.0151). None of the treated mice showed significant weight loss or observable behavioural changes.

These data suggest that expression of an AML-targeting CAR further enhances the antileukemic efficacy of activated NK cells in vivo.

### Proteasome inhibitor mediated sensitization to *CAR*-NK mediated killing is selective for malignant cells and non-toxic to HSC and healthy PBMC

**Fig. 7:**
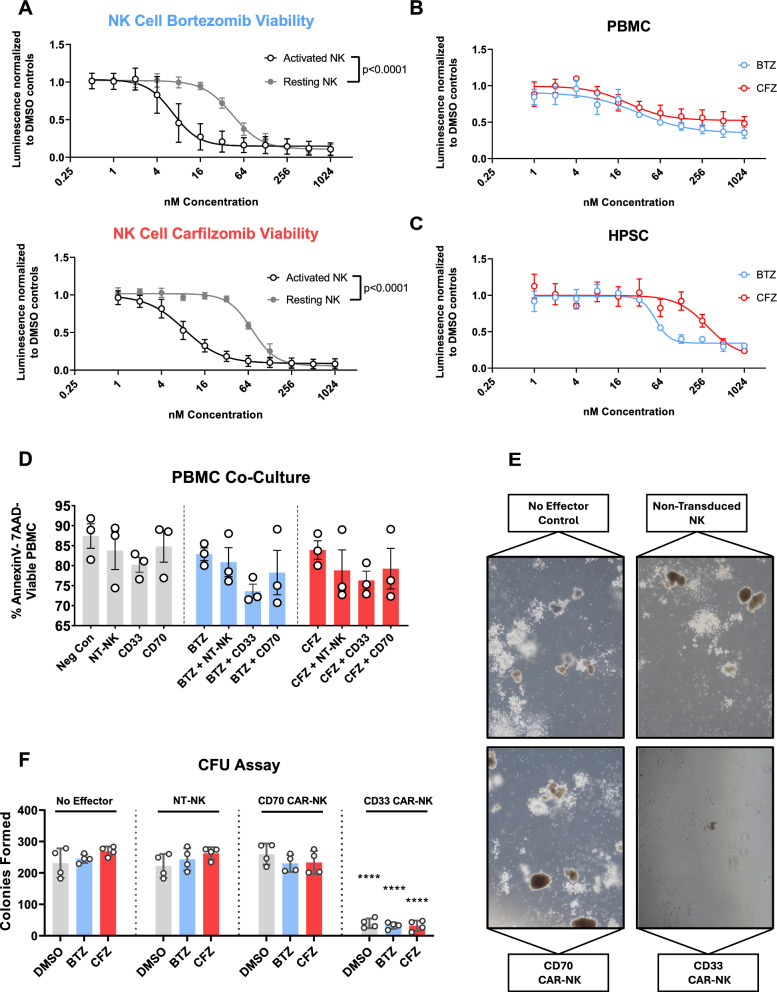
Safety and myelotoxicity of combinatorial therapy with proteasomal inhibitors and CAR-NK effectors. **a** Juxtaposition of IC50 curves of activated and resting NK cells treated with either Bortezomib or Carfilzomib for 24 h. The luminescent signal at each measured dose level is normalised to the corresponding DMSO controls. Each group contains n = 6 matched healthy donors. Statistical comparison of IC50 between sets by Extra-sum-of-squares F test. **b** IC50 curves of PBMCs derived from six different healthy donors following 24-h treatment with Bortezomib (in blue) or Carfilzomib (in red). The luminescent signal at each measured dose level is normalised to the corresponding DMSO controls. Values indicated at each dose level are means of three independent experiments ± S.E.M. **c** IC50 curves of HPSCs following 24-h treatment with Bortezomib (in blue) or Carfilzomib (in red). The luminescent signal at each measured dose level is normalised to the corresponding DMSO controls. Shown are values of a pooled HPSC population derived from n = 3 different donors. Measurements were performed in technical hextuplicate. Values indicated at each dose level are means of three independent experiments ± S.E.M. **d** PBMC from three different healthy donors were pre-treated with Bortezomib, Carfilzomib or a DMSO control for 24 h. Afterwards, they were co-cultured with allogeneic aNK or CAR-NK cells for 24 h. Shown are means of three healthy donors ± S.E.M. **e** Brightfield microscopy images of colonies formed in methylcellulose after 10 days of culture. **f** CD70-CAR NK, CD33-CAR NK, and non-transduced activated NK cells were co-cultured with healthy CD34-positive HPSCs at an E:T ratio of 10:1 for 6 h. The HPSCs were then seeded in 12-well plates in a standard CFU assay. All wells were seeded in technical duplicate with n = 3 different NK cell donors. The number of colonies was determined after 10 days of incubation. Shown are the mean number of colonies formed ± S.E.M. Two independent investigators counted colonies from 2 technical replicates for each condition. Statistical comparison by two-way ANOVA with a post-hoc Dunnett multiple comparison test to the media only control. *P < .05; **P < .01; ***P < .001, ***P < .0001

Having demonstrated that proteasomal inhibition is a highly effective in sensitizing AML cells to NK mediated killing, we sought to determine potential on-target off-tumor toxicity against normal cells of the hematopoietic system. We demonstrate that activated NK cells are significantly more susceptible to proteasome inhibition than their non-stimulated counterparts (Fig. 7A). Furthermore, PBMC and CD34 + hematopoietic progenitor and stem cells (HPSC) are less susceptible to PI-mediated killing, indicated by 10 to 100-fold higher IC50 values compared to AML cells or activated NKs (Fig. 7B–C, Figure S2).

Treating PBMC with increasing doses of PIs induced similar changes with respect to the HLA, death ligand and SAP expression as seen in AML but required higher concentrations of the respective compound (Figure S22). To test the cytotoxicity of the combinatorial therapy against PBMC, we exposed PBMC from four different healthy donors to Bortezomib or Carfilzomib for 24 h and then co-cultured them with allogeneic (CAR-)NK cells. The schematic overview of the experiments is shown in Figure S21A. Unlike the AML cell lines, PBMC were not susceptible to allogeneic NK-mediated killing (Fig. 7D).

As shown in Fig. 7C, HPSC are highly resistant to proteasomal inhibition (IC50_Carfilzomib_ 301,2 nM; IC50_Bortezomib_ 55,0 nM). We developed an in-vitro model to investigate the myelotoxicity of the combination of PI pre-treatment and CAR-NK (Figure S21A). Following pre-treatment of HPSC with Bortezomib, Carfilzomib or a DMSO control for 24 h, we co-cultured them with NK cells at a high effector-to-target ratio before seeding them in a growth-factor containing methylcellulose media. As expected, based on previous results [32, 34], CD33 CAR-NK cells were highly toxic against HPSC, significantly reducing the ability to form colonies (Fig. 7E–F). In contrast, both non-transduced NK controls and the CD70 CAR-NK did not decrease colony formation (Fig. 7F), suggesting that CD33-targeting immunotherapy but not PI pre-treatment in combination with NK-cells in general impairs normal hematopoiesis. PI pre-treatment did not have a significant effect on colony forming unit count (Fig. 7F).

Taken together, PI-mediated toxicity and sensitization to NK-mediated killing are highly dependent on the activation and proliferation status of the target cells. Healthy cells and in particular HPSC are not susceptible to proteasome inhibition at PI doses that are effective against AML cells.

## Discussion

Advanced cellular therapies might improve outcome in AML but the optimal cell platform and potential strategies to enhance their safety and efficacy remain to be determined. In the current study we demonstrate that proteasome inhibitors such as Bortezomib and Carfilzomib sensitize AML cells to NK cell mediated killing by increasing the expression of stress-associated proteins and down-regulating class-I HLA expression.

The adoptive transfer of T-cells genetically modified to express a CAR has demonstrated impressive clinical results in patients with B-cell lymphoma and multiple myeloma [35]. However, the translation of this promising treatment strategy to patients with AML has proven to be challenging [36, 37]. One major obstacle in AML patients has been the extended manufacturing time lapse between leukapheresis and CAR T-cell infusion [5] preventing many patients from being infused with their CART product due to progressive disease. Further, CAR-T treatment of AML patients has been associated with significant toxicity [3]. Off-the-shelf allogeneic NK-cell therapy presents a promising treatment alternative [38, 39]. Previous studies evaluating the safety and efficacy of adoptive NK cell therapy for the treatment of AML have shown inconsistent results [40, 41]. This may be attributable to immune evasion mechanisms commonly observed in AML, such as a reduced expression of stress-associated proteins [15] and a high density of class-I HLA molecules leading to impaired NK cell mediated killing [42]. Proteasome inhibitors have emerged as a highly active treatment strategy for multiple myeloma and mantle cell lymphoma [43]. There is also increasing evidence that targeting the proteasome may be effective against AML, especially when aiming to eliminate AML-associated leukemic stem cells (LSCs) [44–46]. In addition, proteasome inhibition may overcome therapy resistance in AML via EZH2 stabilization [47].

In line with previously reported studies [42, 48], we demonstrate that Bortezomib and Carfilzomib, two different FDA- and EMA-approved PIs, exhibit significant cytotoxicity against AML cell lines and primary patient samples in vitro even at low-nanomolar concentrations. In addition to their cytotoxic effect, PIs modulate the surface expression of various ligands of NK cell receptors such as class-I HLA molecules [17, 49] and stress-associated proteins [50]. Using a panel of AML cell lines, we show that both effects apply in AML, expanding upon the results of preclinical studies in multiple myeloma [17, 49, 51], glioblastoma [52] and breast cancer [53].

Mechanistically, PI treatment induces the accumulation of unfolded proteins in the endoplasmic reticulum (ER) which activates multiple ER-stress-pathways including the Unfolded Protein Response (UPR) pathway [54] leading to attenuation of overall protein synthesis. Thus, the instant reduction in class-I HLA expression that we observed after 24 h is a consequence of the cells´ impaired ability to produce the peptides necessary for stabilizing the HLA complex on the cell surface [55]. On the other hand, our data also revealed a rapid increase of stress-associated protein encoding mRNA within 24 h of PI treatment, but an excessive activation of the ER-stress-pathway may prevent their translation through the PERK-induced phosphorylation of eIF2α [56] potentially causing the delay of stress-associated protein expression. These observed differences may explain contradicting results from previously published studies that attribute the synergy between PI treatment and NK cell therapy in multiple myeloma exclusively to the decrease in class-I HLA [17, 49] or to the elevated stress-associated protein expression [50]. Activated NK cells are susceptible to PI treatment at similar doses as the AML cells. Thus, we established a therapeutic model in which treatment of AML cells with Bortezomib and Carfilzomib precedes the injection of NK or CAR-NK cells, hereby sparing them from PI-mediated cytotoxicity. This approach is feasible to be applied to the treatment of patients because pharmacological studies in humans have shown that Bortezomib and Carfilzomib serum concentrations diminish rapidly after intravenous injection due to absorption into the intracellular compartment and enzymatic degradation, respectively [57–59]. Accordingly, NK cells infused 24 h after treatment with PIs would be exposed to substantially lower concentrations of the drugs than with a simultaneous treatment strategy [57–59].

Recent publications have highlighted the importance of two different, but complementary pathways of NK cell mediated cytotoxicity: the degranulation-dependent and death-ligand dependent pathways [60, 61]. The former requires the formation of a stable immune synapse, followed by microtubule polarization and finally degranulation leading to the release of Granzyme and Perforin that compromises the integrity of the target cell [60, 61]. The latter relies on interactions between death ligands such as FasL and TRAIL on the surface of NK cells and death receptors like Fas and DR4/DR5 on the surface of target cells [60, 61]. We observed enhanced immune conjugate formation between AML and NK cells as well as higher levels of Interferon-γ secretion by NK cells after PI pre-treatment as described previously [50]. However, exposure to Bortezomib and Carfilzomib also sensitized AML cells to NK cell mediated killing by soluble death ligands [53] suggesting that PI treatment enhances both degranulation-dependent and degranulation-independent cytotoxicity.

The standard of care for AML patients ineligible for intensive chemotherapy is formed by Venetoclax, a potent BCL2 inhibitor, in combination with hypomethylating agents such as azacytidine [20]. However, patients with relapse after or refractory to treatment with Venetoclax/HMA carry a dismal prognosis and are in urgent need for innovative treatment strategies [2]. Exhibiting an excellent safety and tolerability profile [62], adoptive NK cell therapy may be a promising strategy for these patients. We found that resistance of AML cell lines to VEN/AZA treatment had little impact on their susceptibility to PI-mediated cytotoxicity. However, the resistant cell lines were less likely to be eliminated by (CAR-)NK cells alone. The anti-leukemic activity of NK cells could be restored through PI pre-treatment, suggesting that combining PI with NK-cell therapy could also be a promising strategy for this difficult-to-treat patient population.

In-vivo, we confirmed the superior anti-leukemic activity of PI pre-treatment combined with subsequent NK-cell infusion. Importantly, this effect was observable even after a single aNK infusion and without additional systemic cytokine support. Interestingly, while Carfilzomib and Bortezomib showed similar effects in-vitro, only Bortezomib combined with aNK induced a substantial antileukemic effect in our murine xenograft model. While this unexpected result is being explored in ongoing experiments, we hypothesize that it may be attributable to pharmacokinetic differences rather than to the superior efficacy of one compound compared to the other. Our in-vitro experiments were performed using the IC50 concentration of each drug for the respective cell line to improve the comparability between both compounds. However, for our in-vivo studies, the PI doses were based on previous published studies [63, 64]. Pharmacokinetic studies of Carfilzomib have shown that low concentrations of the drug inhibit only the beta-5 subunit, while high concentrations can effectively co-inhibit the beta-1/2 proteolytic subunits leading to superior responses in-vitro and in-vivo [65]. This suggests that a distinct threshold dose of Carfilzomib is necessary to obtain the optimal effect and that the dose applied in our model may have been too low, leading to inferior antileukemic activity.

The anti-leukemic efficacy of NK cell therapy after PI pre-treatment can be enhanced further through the expression of an AML directed CAR construct. By testing a CD70- and a CD33-specific CAR we demonstrate that the benefits in antileukemic activity conferred by combinatorial treatment are independent of the target antigen.

In addition to the overall survival benefit for treated mice, CAR-NK cells in combination with PI pre-treatment were well tolerated, suggesting that this combinatorial treatment approach is similarly safe and tolerable as CAR NK-cells alone. We acknowledge that PI treatment followed by a single CAR-NK cell infusion was not curative in any of our in-vivo models, most likely owing to the short survival of unsupported NK cells in vivo. Achieving more durable responses will likely require the addition of an endogenous stimulating cassette [66], multiple NK infusions [67] or a combination thereof.

## Conclusion

Our study provides evidence that pre-treatment of AML cells with proteasome inhibitors in combination with subsequent CAR-NK cell application is a novel and promising therapeutic strategy for patients with AML. We demonstrate the immunomodulatory effects of PI treatment on AML cells which sensitize them to NK mediated cytotoxicity and showcase the synergistic antileukemic effects of off-the-shelf, ex-vivo activated allogeneic NK cells in vitro and in vivo. Finally, we use two different novel CAR constructs to further enhance NK cell activity against AML in vitro and in vivo.

## Materials and methods

### Retroviral vector production

The process of producing retroviral vectors and transducing T-cells followed previously established methods [32]. In short, HEK293T cells were transiently transfected with two packaging plasmids (PeqPam, RD114) along with the SFG vector containing the CAR construct, using GeneJuice (Merck Millipore, Billerica, MA). Retroviral supernatant was collected after 48 and 72 h and stored at − 80C. 48-h and 72-h supernatants were mixed in a one-to-one ratio prior to transduction.

### NK cell isolation, expansion and *CAR*-NK generation

Peripheral blood mononuclear cells were obtained from peripheral blood by standard Ficoll-paque density gradient centrifugation, and T-cells were depleted via LD MACS columns (Miltenyi, Bergisch Gladbach, Germany) and CD3 microbeads (Miltenyi, Bergisch Gladbach, Germany) in accordance with the manufacturer's instructions. Subsequently, NK cells were activated by four-day co-culture with irradiated feeder cells in a G-Rex 10 vessel (Wilson Wolf Manufacturing, St. Paul, MN) prior to being transduced in non-TC-treated, 24-well plates coated with RetroNectin (Takara Bio, Kusatsu, Japan). The NK cell expansion media consisted of 90% SCGM (CellGenix, Freiburg, Germany) supplemented with 10% Gibco™ Fetal Bovine Serum (ThermoFisher, Waltham, MA), 1% penicillin/streptomycin antibiotic mix (ThermoFisher, Waltham, MA) and 2 mM L-glutamine (Invitrogen, Waltham, MA). To support NK cell expansion, rhIL-15 (Peprotech, Hamburg, Germany) was supplemented at regular intervals to a final concentration of 10 ng/ml. The growing NK cell cultures were split on days 7, 10 and 14 of production. CAR-NK transduction efficiency was measured on day 10 of production via flow cytometry as described previously [32]. The NK cell percentage purity of the cultures was measured at regular intervals via flow cytometry. Functional assays were performed between days 14 and 17 of production.

### Cell lines

The K562 CS feeder cell line was a kind gift from Prof. Rooney (Baylor College of Medicine, Houston, TX). The HL-60, Molm-13, OCI-AML2, U937, HEL, IMS-M2, KG1-a, THP1, MV4-11, K562, Molp-8 and RPMI-8226 cell lines were obtained from the DSMZ (German Collection of Microorganisms and Cell Cultures, Braunschweig, Germany). All cell lines were authenticated through the DSMZ prior to their use in experiments. All cell lines were subjected to regular mycoplasma contamination testing using a PCR Mycoplasma Test Kit I/C (PromoCell, Heidelberg, Germany). All cell lines were maintained in RPMI-1640 supplemented with 10% HI-FBS and 2 mM L-glutamine as well as 1% penicillin/streptomycin (ThermoFisher, Waltham, MA) in a humidified atmosphere containing 5% CO2 at 37 °C. The azacitidine/venetoclax resistant cell lines Molm-13, HL-60 and OCI-AML2 were a kind gift from Prof. Müller-Tidow (University Clinic Heidelberg, Heidelberg, Germany). The process of their conditioning is described in detail in [68]. A final concentration of 300 nM venetoclax (Selleck Chemicals, Cologne, Germany) and 1 µM azacitidine (Selleck Chemicals, Cologne, Germany) was added weekly to maintain the resistance of these cell lines. The cells were washed thoroughly of azacitidine and venetoclax immediately prior to commencing experiments.

### Primary AML sample culture

Primary AML samples were sourced from the biobank of the University Clinic Heidelberg and maintained in serum-free Stemspan media (StemCell Technologies, Vancouver, Canada) supplemented as described in [69] with 1 µM UM729, 20 ng/ml IL3, 20 ng/ml FLT3-L, 20 ng/l SCF, 20 ng/ml IL-6, 50 ng/ml TPO and 1% penicillin/streptomycin (ThermoFisher, Waltham, MA) for the duration of the experiments.

### HPSC culture and CFU assay

HPSCs were isolated via MACS. After thawing, the HPSCs were controlled for CD34 + purity and their expression of CD33 and CD70 was measured via flow cytometry. HPSCs were kept in culture for 24 h after thawing. Colony formation assays were performed with H4434 methylcellulose from StemCell Technologies according to the manufacturer´s instructions. Briefly, the contents of co-cultures containing 2.000 viable HPSC were transferred into 2 ml of methylcellulose media and plated into 12-well tissue-culture treated plates. After 10 days of culture, the colonies formed were counted and visually recorded using a CKX53 microscope (Olympus LS).

### CellTiter Glo viability assay and IC50 calculation

Cells were dispensed in 96-well flat-bottom, white, chimney-well plates in 100 µl of IMDM (Invitrogen, Waltham, MA) supplemented with 10% HI-FBS (ThermoFisher, Waltham, MA), then exposed to a serial dilution of Bortezomib or Carfilzomib in DMSO, or a DMSO-only control. After 24 h, total cell viability was measured using the CellTiter Glo luminescent viability assay (Promega, Fitchburg, USA) on a Tecan Spark microplate reader (Tecan, Männedorf, Switzerland), following the manufacturer's instructions. The absolute luminescence values were normalized to those of the DMSO controls. The IC50 values for each cell line or primary sample were calculated in GraphPad Prism7 using the nonlinear fit of [Inhibitor] versus response (four parameters). All measurements were performed in technical triplicate unless specifically stated otherwise.

### Flow cytometry

The fluorochrome-conjugated anti-human antibodies were purchased from BD Biosciences (San Jose, CA) or Biolegend (San Diego, CA). Biotinylated protein L and fluorochrome-conjugated streptavidin for CD33 CAR detection were purchased from Biolegend (San Diego, CA). A complete list of all antibodies used in this work, including catalog numbers, is provided in the supplementary methods section (Figure S4). Flow cytometric data were acquired on the BD-LSR II (BD Bioscience, San Jose, CA) and BD-Celesta (BD Bioscience, San Jose, CA) flow cytometers using the high-throughput sampling module. Flow cytometric data was analyzed with FlowJo, version 10 (Tree Star, Ashland, OR).

### In vitro NK cell functional analysis

Following 14 days of culture, NK cells were washed and resuspended in RPMI-1640 supplemented with 10% FBS. To easily distinguish target from effector cells, AML cell lines were tagged with CellTracker Green CMFDA (ThermoFisher, Waltham, MA) at a concentration of 1 µM following the manufacturer's instructions. After the fluorescent tagging step, the AML cell lines were incubated with Bortezomib or Carfilzomib at the determined IC50 dose, or a DMSO-control for 24 h as indicated. Following this pre-treatment, the target cells were thoroughly washed, and viable cell counts were determined using CountBright absolute counting beads (ThermoFisher, Waltham MA, USA) on a BD-Celesta flow cytometer before seeding in plates in the presence of NK cells.

### Co-culture assay

AML cell lines were pre-treated for 24 h with DMSO or IC50 Bortezomib or Carfilzomib, respectively. The cells were then washed thoroughly and tagged with a 1 μM concentration of CellTracker Green, following the manufacturer´s instructions. After determining the counts and viability of the AML cells in each group via flow cytometry, 25,000 viable tumor cells in 100 μl of complete media were dispensed in each well of a 96-well flat bottom TC-plate. Following a 24-h incubation the contents of each well were harvested, mixed with counting beads and 7-AAD, then measured using the high-throughput sampling module attached to a BD Celesta flow cytometer. For co-culture assays performed in a 96-well plate format, 2.5E4 viable AML cells were dispensed per well. NK cells were added to the AML cells at a ratio of 2-to-1, 1-to-1 or 1-to-2 as stated in the corresponding figure legend. Twenty-four hours after the start of co-culture, cells were washed, CountBright beads (ThermoFisher, Waltham MA, USA) were added, and the co-culture was stained with 7-AAD (Biolegend, San Diego, CA). The co-cultures were acquired on a BD-Celesta flow cytometer (BD Bioscience, San Jose, CA) using a high-throughput sampling unit. The absolute cell counts per well were determined by normalizing 7AAD_neg_ viable cell counts to absolute counting beads in accordance with the manufacturer´s instructions.

### Primary AML sample co-cultures

Prior to starting the co-culture, six primary AML samples were fluorescently tagged using CellTracker Green CMFDA (ThermoFisher, Waltham MA, USA) as described above, then pre-treated with a fixed concentration of 1 nM Bortezomib (Selleck Chemicals, Cologne, Germany) or 2 nM Carfilzomib (Selleck Chemicals, Cologne, Germany). To maintain AML cell viability and prevent in vitro differentiation during co-culture, both the AML cells and the NK cell effectors were washed and resuspended in serum-free primary Stemspan AML media supplemented as described above. The primary AML sample co-cultures were performed in a 384-well format. The viable cell count dispensed per well was adjusted to 1,25E4 to better fit the smaller wells. Staining and analysis were performed in the same fashion as the AML cell line co-cultures.

### Stable conjugate formation assay

Stable conjugate formation was measured as described previously [70] and shown schematically (Figure S8B). Briefly, AML cell lines were fluorescently tagged and pre-treated with proteasome inhibitors at IC50 concentrations or a DMSO control for 24 h. Following this, AML cells were washed, counted, and mixed with NK cells at a 1-to-1 ratio in 5 ml round-bottom polypropylene FACS tubes. Five minutes later, the co-cultures were fixed with ice-cold 70% ethanol while vortexing vigorously, before being put on ice and immediately proceeding to flow cytometric analysis.

### Interferon-γ ELISA

Following 24-h co-culture, 100 µl of supernatant were collected from each well and stored at -80 C. After thawing the supernatants, the concentration of Interferon-γ was measured using a standard Human Duo-Set ELISA kit and Ancillary Reagent kit II (R&D Systems, Minneapolis, USA) following the manufacturer's instructions.

### Concurrent combinatorial treatment assay

ZsGreen-expressing U-937, Molm-13 and HL-60 AML cells were seeded in technical triplicate in flat-bottom 96-well TC plates. The culture media contained the respective IC50 concentration of Bortezomib or Carfilzomib, or a DMSO control. Concurrently, NK cells were seeded at a ratio of 1-to-1. After 48 h of co-culture, the first row of technical replicates was harvested, stained and the absolute counts of NK- and tumor cells were measured via flow cytometry. In all remaining wells, 100 µl of supernatant was removed before being exchanged with 100 µl of cell media containing proteasome inhibitors and a further 2,5E4 viable AML cells. The cells were then left in co-culture for a further 48 h before a second measurement was performed in analog to the first.

### Western blot

Cell pellets were lysed in ice-cold RIPA-buffer containing 1X HALT protease inhibitor cocktail (ThermoFisher, Waltham, MA, USA). The protein concentration of the lysates was determined with a Pierce BCA Protein Assay Kit (ThermoFisher, Waltham, MA, USA) and measured on a TecanSpark microplate reader (Tecan, Männedorf, Switzerland). The protein samples were separated via SDS-PAGE gel electrophoresis and blotted on a nitrocellulose membrane as described previously [71]. 10 µg of total protein were pipetted in each pocket of the gel comb. The membranes were blocked with 1% BSA (ThermoFisher, Waltham, MA, USA) in TBS-T and stained overnight with the primary antibodies listed in supplementary Figure S4 followed by a stain with a mouse anti-rabbit IgG HRP-conjugate and chemiluminescent image capture.

### Dynamic NFkB activity monitoring using the Jurkat TPR

The Jurkat triple parameter reporter cell line (Jurkat_TPR_) [72] was a kind gift from the Steinberger lab (University Clinic Vienna, Austria). The cells were maintained in RPMI-1640 supplemented with 10% HI-FBS and 2 mM l-glutamine as well as 1% penicillin/streptomycin (ThermoFisher, Waltham, MA) in a humidified atmosphere containing 5% CO2 at 37 °C. Jurkat_TPR_ cells were activated using the Cell Activation Cocktail at a 1:500 dilution (without Brefeldin A) (Biolegend, San Diego, CA) for 12 h before Bortezomib, Carfilzomib or a DMSO control were added. Twelve hours later, the cells were washed and stained on ice prior to acquisition on a BD-LSR II flow cytometer.

### Two-step RT-qPCR

Wild-type Molm-13 cells were treated with a serial dilution of Bortezomib for 24 h before lysis and RNA extraction using a commercial silica-column based kit (RNeasy Mini) (Qiagen). After extraction, the concentration and purity of the extracts were measured using a NanoDrop device. Following that, cDNA was synthesized using a PrimeScript RT-PCR kit (Takara Bio, Kusatsu, Japan) and random hexamer primers. qPCR was then performed in technical triplicate using a SYBR Green PCR Master Mix (ThermoFisher). All values were normalized to 18S rRNA as a housekeeping gene, then the experimental samples under proteasome inhibition were normalized to the DMSO controls to show transcript fold-change under proteasomal inhibition.

### Colony forming unit assay

The methylcellulose colony forming unit assay was performed using MethoCult H4434 (StemCell Technologies) according to the manufacturer’s instructions. Briefly, CD34_pos_ human stem-and-progenitor cells (HPSC) were isolated via MACS. A total of 2,000 viable HPSC were dispensed per well of a 96-well U-bottom tissue culture treated plate and co-cultured with 20,000 viable (CAR-) NK cells.

### Xenograft models of AML, bioluminescent imaging

Six-to-twelve-week-old NOD-*Prkdc*^*scid*^*-IL2rg*^*Tm1*^/Rj (NSG) (NSG) mice were transferred from the DKFZ animal facility or sourced from internal breeding at the Interfacultary Biomedical Research Facility at the University of Heidelberg (IBF), as available. The animals were maintained under pathogen free conditions in ventilated cages at the IBF animal facility. The animal experiments described were approved by the Animal Welfare Committee of the Karlsruhe regional council and performed under approval number 35-9185.81/G291/21. The NSG mice received intravenous injections of tumor cells and NK cells via the tail vein after infrared light illumination. Proteasome inhibitors were injected in the tail vein at the doses stated in the relevant results section. Prior to injection, the proteasome inhibitors Bortezomib and Carfilzomib were mixed with PEG300 (SelleckChem, Cologne, Germany) and Tween80 (SelleckChem, Cologne, Germany) at the ratio recommended for in vivo use by the company. All AML cell lines used in the animal experiments were stably transduced with a construct encoding zsGreen and Click Beetle Green to allow for their flow-cytometric and bioluminescent tracking, respectively. The AML-cell burden was monitored by bioluminescent imaging (BLI; photons per second per cm2 spectral radiance), using the in-house IBF Xenogen In Vivo Imaging System (IVIS; Caliper Life Sciences, Hopkinton, MA) ten minutes after the intraperitoneal injection of D-Luciferin substrate (PerkinElmer, Waltham, MA).

### Calculating synergy

Synergy was calculated using Jin’s modified Bürgi formula as described previously [27, 28].

$$Combinatorial\, Index\, \left(q\right)\,=\,\frac{Observed \,Effect}{Predicted \,Effect}$$

Synergy is defined as a combinatorial index (q) greater than 1. A combinatorial index of 1 indicates additivity and a combinatorial index of less than 1 indicates anti-synergy.

To calculate the combinatorial index, we first defined the effect being measured. Our parameter of interest is the antileukemic potency of the proposed treatment and the surrogate marker is the viability loss of the AML cells.

Viability loss was defined as follows:

$$Viability\; loss = \frac{{\% Surviving\; cells \,\left( {treatment} \right)}}{{\% Surviving\; cells\, \left( {control} \right)}} {-} 1$$

The predicted viability loss was defined as the theoretical loss of viability caused by the antileukemic efficacy of NK cells and proteasome inhibitors if these were purely additive.

Thus, the final equation for synergy calculations was:

$$\tiny Combinatorial\, Index \,\left(q\right)\,=\, \frac{Obsered \,viability \,loss\, \left(Combination \,Treatment \,A+B\right)}{\left(Viability \,loss \,\left(Treatment \,A\right)*Viability \,loss \,\left(Treatment \,B\right)\right)}$$

$$Combinatorial\, Index\, (q)\,=\,1\, Additive\, effect \,only$$

$$Combinatorial \,Index\, (q)\,<1\, Antagonism$$

$$Combinatorial \,Index\, (q)\,>1\, Synergy$$

Statistical testing was done by calculating the Combinatorial Index as described above and performing a one-sample t-test against the hypothetical value of 1.0.

### Statistical analysis and data visualization

Statistical analysis was performed using Prism 7 (GraphPad Software, San Diego, CA). The 2-tailed Student t test was used for comparisons between two groups. When comparing the means of more than two groups, a post-hoc multiple comparison analysis was performed. Dunnett’s multiple comparison correction was used when comparing multiple groups to a control group. Tukey’s multiple comparison correction was used when comparing the means of each group with the means of every other group. Survival was compared using log-rank (Mantel-Cox) tests after constructing Kaplan–Meier curves. Data was visualized using Prism 7 (GraphPad Software, San Diego, CA). Unless explicitly stated otherwise, all error bars indicate the means and the standard error of the mean (S.E.M).

## Supplementary Information

1.

## Data Availability

The datasets used and/or analysed during the current study are available from the corresponding author on reasonable request.
