# Supplementary material for: Proteasome inhibition enhances the anti-leukemic efficacy of chimeric antigen receptor (CAR) expressing NK cells against acute myeloid leukemia
Source: J Hematol Oncol. 2024 Sep 16;17:85. doi: 10.1186/s13045-024-01604-y (PMC11406742; doi:10.1186/s13045-024-01604-y)

Supplementary Figure Legends:

Figure S1 IC50 curves of all cell lines included in the titration experiment. IC50 curves following 24-hour treatment with Bortezomib or Carfilzomib. The luminescent signal at each measured dose level is normalised to the corresponding DMSO controls. Values indicated at each dose level are means of three independent experiments ± S.E.M.

Figure S2 IC50 values of all cell lines tested, as well as activated NK cells. Values given are means of three independent experiments. Relative resistance ranks are assigned to each cell type in descending order of Carfilzomib resistance. The order of resistance is different for Bortezomib, as indicated by the out-of-order ranks in the leftmost column. Activated primary NK cells were derived from eight different healthy donors.

Figure S3 Robust expansion and distinct phenotype changes induced by 14-day expansion protocol. a Flow-cytometric verification of the expression of all upregulated parameters on K562-CS feeder cells, juxtaposed to wild-type K562 controls. b Proliferation curve of eight different healthy donors throughout the course of 14-day production. Line follows the mean; each dot is indicative of a different healthy donor. c Representative histograms outlining the rise of NKG2D expression on the surface of NK cells derived from donor #3 throughout the course of production. d Diagrams outlining the development of nine different functionally relevant NK cell surface proteins throughout the course of production. Line follows the mean at each measurement; each dot is indicative of a different healthy donor. e Heatmap representing the relative expression of all measured parameters throughout the course of production. All values are normalized to the gMFI measured on circulating peripheral blood NK cells before the start of the production.

Figure S4 List of all antibodies, reagents and qPCR Primers used in the experiments reported in this manuscript.

Figure S5 Proteasome inhibition rapidly and steeply inhibits constitutive NFkB signaling in AML. a Proteasome inhibition lowers NFkB signal intensity in PMA/Ionomycin activated Jurkat TPR. On the left are two graphs showing gMFI of the GFP and CFP reporter proteins. Solid lines indicate the values measured in PMA/Ionomycin-activated Jurkat TPR under proteasome inhibition. Interrupted lines indicate the values measured in non-activated TPR Jurkat cells under proteasome inhibition. High concentrations of Carfilzomib lower both NFkB and NFAT signal intensities down to the level observed in resting cells. Bortezomib exerts a strong effect on NFkB reporter intensity, but not NFAT reporter intensity. b Example histograms for all three reporters under rising concentrations of each proteasome inhibitor.

Figure S6 Effects of Cytarabine and Daunorubicin on the Molm-13 surface proteome. A IC50 curves following 24-hour treatment of wild-type Molm-13 cells with Cytarabine or Daunorubicin. The luminescent signal at each measured dose level is normalised to the corresponding DMSO controls. Values indicated at each dose level are means of three independent experiments ± S.E.M. The IC50 values and their confidence intervals can be found in the supplementary Figure S2. B Heatmap of the relative quantitative expression of select proteins on the surface of wild-type Molm-13 cells under the influence of Cytarabine or Daunorubicin. The values shown represent the gMFI of each protein on viable cells after normalisation to DMSO-controls.

Figure S7 Proteasomal inhibition causes an immunogenic cell death and lowers the expression of PD-L1/L2 in a dose-dependent manner. A Eleven different AML cell lines were treated with Bortezomib, Carfilzomib or a DMSO control for 24 hours. The surface expression of Calreticulin was then measured via flow cytometry. Values shown are gMFI of each cell line normalized to the respective DMSO control. Statistical comparison by one-way ANOVA followed by post-hoc analysis and Dunnett´s multiple comparison correction with the DMSO group serving as control *P < .05; **P < .01; ***P < .001, ***P < .0001 b Wild-type Molm-13 cells were treated with rising concentrations of Bortezomib, Carfilzomib or a DMSO control for 24 hours. The surface expression of PD-L1 and PD-L2 was quantified via flow cytometry.

Figure S8 Representative dot-plot of results shown in Figure 2A. Example flow cytometry plots of aNK co-cultures shown in Figure 2A. Gate shows viable, CellTrackerGreen positive fluorescently tagged AML cells. Representative plots of U-937 targets and aNK derived from four different healthy donors.

Figure S9 Stable conjugate formation assay shows stronger immune synapse formation after proteasome-inhibitory pre-treatment. a Short summary of experimental setup as described in Molm-13 cells were transduced and sorted for the stable expression of zsGreen, a GFP-derived fluorescent protein. Molm-13.zsG cells were mixed and co-cultured for a short period of time with aNK cells in 5-ml round bottom polypropylene FACS tubes. Created with BioRender.com b Representative dot-plots of stable conjugate formation. Results from aNK derived from three different healthy donors are shown side-by-side.

Figure S10 Proteasome-inhibitor pre-treatment sensitizes AML cell lines to apoptosis induced by soluble death ligands. a Setup of death ligand apoptosis assay. Eleven different AML cell lines were pre-treated with DMSO or Bortezomib/Carfilzomib at IC50 for 24 hours. The cells were then washed thoroughly and 25,000 viable tumor cells in 100µl of complete media were dispensed in each well of a 96-well flat bottom TC-plate. Following this, 100µl of complete media containing DMSO or TRAIL/FasL was added to each well. After a 24-hour incubation, all cells were harvested and stained using an Annexin-V staining kit from Biolegend. Graphics were created with Biorender.com b Representative dot-plots of KG1-a cells under death ligand treatment. Viable cells were defined as 7AADneg AnnexinV_{neg}; apoptotic cells were defined as 7AADpos AnnexinV_{neg}; dead cells were defined as 7AADpos AnnexinV_{pos}. c Heatmap depicting AML cell viability at the end of the death ligand assay. Results represent the mean viability of three independent experiments. d-e Predicted and observed cytotoxicity of the combinatorial treatment with soluble death ligands and PIs. Statistical comparison by unpaired Student t-test.*P < .05; **P < .01; ***P < .001, ***P < .0001.

Figure S11 Blocking HLA-ABC on wild-type Molm-13 enhances NK-mediated toxicity, but blocking NKG2D is not sufficient to hamper NK cell killing. a Experimental setup of HLA-blocking experiment. Molm-13 cells were incubated with 10 µg/ml of an anti-HLA-ABC monoclonal antibody or a mouse anti-IgG control antibody for one hour, then washed thoroughly and put into co-culture with aNK cells. Created with BioRender.com b Experimental setup of the NKG2D blocking experiment. NK cells were incubated with 10 µg/ml of an anti-NKG2D monoclonal antibody or a mouse anti-IgG control antibody for one hour before being washed and co-cultured with Molm-13 cells. Created with BioRender.com f Viable Molm-13 cells after blocking and aNK co-culture. Results are represented as means ± S.E.M. Statistical comparison by one-way ANOVA followed by post-hoc analysis and Dunnett´s multiple comparison correction with the untreated group serving as control d Black-and-white heatmap showing the baseline expression of different proteins on eleven AML cell lines. Values shown represent the mean gMFI of three independent experiments. *P < .05; **P < .01; ***P < .001, ***P < .0001.

Figure S12 Proteasome-inhibitory pre-treatment combined with allogeneic NK cell infusion is well tolerated while showing strong antileukemic efficacy in vivo. a Luminescence-overlay images of all animals throughout the course of the experiment. b Radiance scales at each imaging time point. c Weight development throughout the experiment. The time course of each individual animal has been plotted separately.

Figure S13 Engineering peripheral-blood derived NK cells with two different CAR constructs targeting AML. a CAR-NK production protocol. As in the production of aNK cells, the first four days are comprised of a co-incubation with irradiated K-562 CS feeder cells at a ratio of 1 NK cell for each 5 feeder cells. The initial activation step is performed in G-Rex 10 vessels, allowing for superior seeding density and a high media volume. Because of this, the cells can be left to incubate without the necessity for changing their media, thus not disturbing the natural homotypical activation contacts during their growth phase. On day 4 of production, the growing NK cell culture is transduced by standard spinoculation in 24-well non-tissue culture treated plates coated with Retronectin. The cultures are then split on days seven, ten and fourteen before being harvested for functional assays. Created with BioRender.com. b Schematic diagrams of the structures of the two CAR constructs. The CD33 CAR is of the third generation and comprises an scFv domain, a flexible linker, CD8-derived transmembrane domain, two co-stimulatory domains as well as a CD3z trigger domain. The CD70 ligand-based CAR consists of a full-length CD27 fused to a CD3z domain as described in detail in [32]. A truncated form of CD19 is interspaced by an IRES sequences and expressed downstream of the CAR itself. This is to allow for the detection of transduced cells, due to the endogenous CD27 expression upon NK cells. Due to the CD70 expression of activated NK cells, the production protocol for CD70 CAR-NK included the addition of Dasatinib to a final concentration of 100ng/ml on days 4, 7 and 10 of the production cycle in order to prevent fratricide. CD70 CAR-NK cells were thoroughly rinsed of Dasatinib 24 hours before the start of any functional assay. c Representative flow-cytometric histograms of the transduction efficiency of four healthy donors. CD33 CAR-NK on the left, CD70 CAR-NKon the right. Biological negative control included under each half-overlaid histogram stack, depicted in grey.

Figure S14 Target antigen expression and LSC% of six primary AML samples. a Table containing LSC% and CD33/CD70 expression percentages and gMFI for the six primary AML samples used in Figure 4. The gates for CD33 or CD70 positivity were set based on FMO-controls of each primary sample. b Representative flow-cytometric histograms of the target antigen expression on each AML sample. CD33 expression on the left, CD70 expression on the right. c Comparison of aNK, CD33 CAR-NK and CD70 CAR-NK cell cytotoxicity against six primary AML samples. Depicted are the viable AML cell counts, normalized to DMSO-only control wells. The experiment was performed with four different healthy effector cell donors, all wells without effectors were seeded in technical triplicate. Statistical comparison by one-way ANOVA followed by post-hoc analysis and Dunnett´s multiple comparison correction *P < .05; **P < .01; ***P < .001, ***P < .0001.

Figure S15 Azacitidine/Venetoclax resistant cell lines are susceptible to proteasome-inhibitory pre-treatment and NK-mediated killing. a Schematic representation of the experimental setup. Graphics were created using Biorender. b Representative dot-plots Molm-13 Aza/Ven Res cells against aNK cells derived from four different healthy donors. c Viable Molm-13WT, Molm-13Res, HL-60WT and HL-60Res cell counts after co-culture with aNK or CAR-NK cells. The total viable cell count per well was determined through normalization to absolute counting beads. d Viable AML cell count of after co-culture with aNK, CD33 CAR-NK or CD70 CAR-NK. The AML cells were only pre-treated with a DMSO control, not PIs. N = 4 healthy donors. Statistical comparison by One-way ANOVA followed by post-hoc analysis and Dunnett´s

multiple comparison correction e Viable AML cell count after proteasome inhibitor pre-treatment co-culture. Results from aNK, CD33 CAR-NK and CD70 CAR-NK co-cultures are pooled in each column. N=4 healthy donors. Statistical comparison by one-way ANOVA followed by post-hoc analysis and Dunnett´s multiple comparison correction *P < .05; **P < .01; ***P < .001, ***P < .0001.

Figure S16 Concurrent treatment with PI and NK cells shows short-term benefit in tumor control at the cost of effector cell viability and proliferation. a Schematic representation of the experimental setup. Three AML cell lines modified for the stable expression of zsGreen were co-cultured with NK cells at an effector-target ratio of one-to-two, with 25,000 NK cells and 50,000 AML cells. The media of each well contained Bortezomib/Carfilzomib to a final concentration equal to the IC50 of the respective tumor cell line, or a DMSO control. After 48 hours of co-incubation, the first technical replicate seeded was harvested, stained and the viable cell counts measured on a flow cytometer. The other technical replicates seeded were stimulated for a second time with tumor cells, then left to co-incubate for an additional 48 hours. Graphics were created using BioRender.com b Representative dot-plots of HL-60 Aza/Ven Res cells co-cultured with CD33 CAR-NK. The first row shows the results after a 48-hour incubation period, the second after 96 hours. The left column shows the time progression of DMSO-controls, Carfilzomib concurrent treatment shown on the right. The marginal benefits in tumor control after two days of co-culture are offset by a lower viable effector count. After 96 hours, the Carfilzomib concurrent treatment group displays higher tumor cell counts. c Heatmaps depicting co-culture index values over the course of the concurrent treatment experiment. The co-culture index is a score meant to summarize the two parameters of tumor cell proliferation and effector cell proliferation in a single numerical value. It is calculated by subtracting the tumor cell fold-change relative to the initially seeded counts from the NK cell fold-change relative to the initially seeded counts. A high, positive co-culture index demonstrates strong NK cell proliferation and tumor cell elimination. Inversely, low or negative co-culture index values denote low NK cell proliferation, uncontrolled tumor cell growth or both. The results shown were performed with NK cells derived from four different healthy donors.

Figure S17 Concurrent treatment with PI and NK cells significantly reduces NK cell viability. a Heatmaps depicting viable NK cell counts after 48 or 96 hours of concurrent treatment with PI. NK cells. b Heatmaps showing viable AML cell counts after 48 or 96 hours of concurrent treatment with PI. c Comparison of viable NK cell counts as well as tumor cell counts during concurrent treatment. Statistical comparison by one-way ANOVA followed by post-hoc analysis and Dunnett´s multiple comparison correction *P < .05; **P < .01; ***P < .001, ***P < .0001.

Figure S18 Short-term proteasome inhibition does not prevent NK-mediated T-cell activation. a Schematic representation of co-culture setup. PBMC from four different healthy donors were tagged with CellTracker Green and distributed in four different co-culture conditions – PBMC alone, PBMC and HL60, PBMC and allogeneic NK cells or PBMC, allogeneic NK cells and HL-60 together. The PBMC-only group served as a negative control. The supernatant contained Bortezomib, Carfilzomib or a DMSO control. After 24 hours of co-culture with or without proteasome inhibition, the expression of CD69 on viable, CTG-tagged CD3+ T-cells was measured via flow cytometry. Figure created with BioRender.com. b CD69 gMFI on T-cells is upregulated upon co-culture with NK-cells and tumor targets. Exposure to proteasome inhibitors does not have a statistically significant effect on the upregulation of CD69. Statistical comparison by two-way ANOVA. *P < .05; **P < .01; ***P < .001, ***P < .0001.

Figure S19 CD33-CAR-NK cells outperform aNK controls as and further boost the efficacy of PI pre-treatment combination treatment. a Short summary of the experimental setup. On day -5, AML

engraftment was initiated through the intravenous injection of 1E6 U-937 cells. Bortezomib or a vehicle-only was injected intravenously four days later. Non-transduced, feeder-cell activated NK cells or CD33 CAR-NK were injected intravenously 24 hours after the single-dose proteasome inhibitor treatment. BLI was performed immediately prior to NK cell injection and repeated weekly thereafter. Figure created with Biorender.com. b Luminescence-overlay images of all animals throughout the course of the experiment. c Radiance scales at each imaging time point.

Figure S20 CD70-CAR-NK cells outperform aNK controls and further boost the efficacy of PI pre-treatment combination treatment. a Short summary of the experimental setup. On day -5, AML engraftment was initiated through the intravenous injection of 1E6 Molm-13 cells. Bortezomib or a vehicle-only was injected intravenously four days later. Non-transduced, feeder-cell activated NK cells or CD70 CAR-NK were injected intravenously 24 hours after the single-dose proteasome inhibitor treatment. BLI was performed immediately prior to NK cell injection and repeated weekly thereafter. Created with Biorender.com b Luminescence-overlay images of all animals throughout the course of the experiment. Radiance scales at each imaging time point are shown on the right.

Figure S21 Co-culture setups of safety experiments with PBMC and HPSC. a Schematic representation of co-culture setup. PBMC from four different healthy donors were tagged with CellTracker Green and treated with Bortezomib, Carfilzomib or a DMSO control for 24 hours. After 24 hours of proteasome inhibitory pre-treatment, viable cells were counted and distributed into co-cultures with or without allogeneic NK cells at an effector to target ratio of 1:1 with 50,000 viable cells per well. After 24 hours of co-culture, cells were stained with an Annexin V kit. The primary endpoint was the percentage of viable CTG-tagged PBMC between the different co-culture conditions. Figure created with BioRender.com. b Schematic representation of HPSC co-culture and CFU assay. CD34+ HPSC were isolated from a single donor via MACS and frozen for later use. After thawing, the HPSC were treated with Bortezomib, Carfilzomib or a DMSO control for 24 hours. They were then counted and distributed into U-bottom 96-well plates for co-culture with NK cells. Activated non-transduced NK, CD33 CAR-NK or CD70 CAR-NK from four different healthy donors served as effectors. The co-culture was performed at a high effector-to-target ratio of 10:1 with 20.000 NK cells and 2.000 viable HPSC in each well. After 6 hours, the contents of each well were transferred into 2ml of enriched methylcellulose media and plated in TC-treated 12-well plates in technical duplicate. The number of colonies formed was counted after 10 days of culture. c Flow cytometric measurements of CD33 and CD70 expression on HPSC. The gates were set using FMO controls.

Figure S22 Proteasome inhibitor induced phenotype changes in PBMC. Heatmaps of the relative quantitative expression of select proteins on the surface of PBMC treated with rising doses of Bortezomib and Carfilzomib. Measurements represent gMFI of each protein on viable cells after normalisation to DMSO-controls. N=3 healthy donors. Values shown represent the means of the three donors used.

Supplementary Figure S1

A

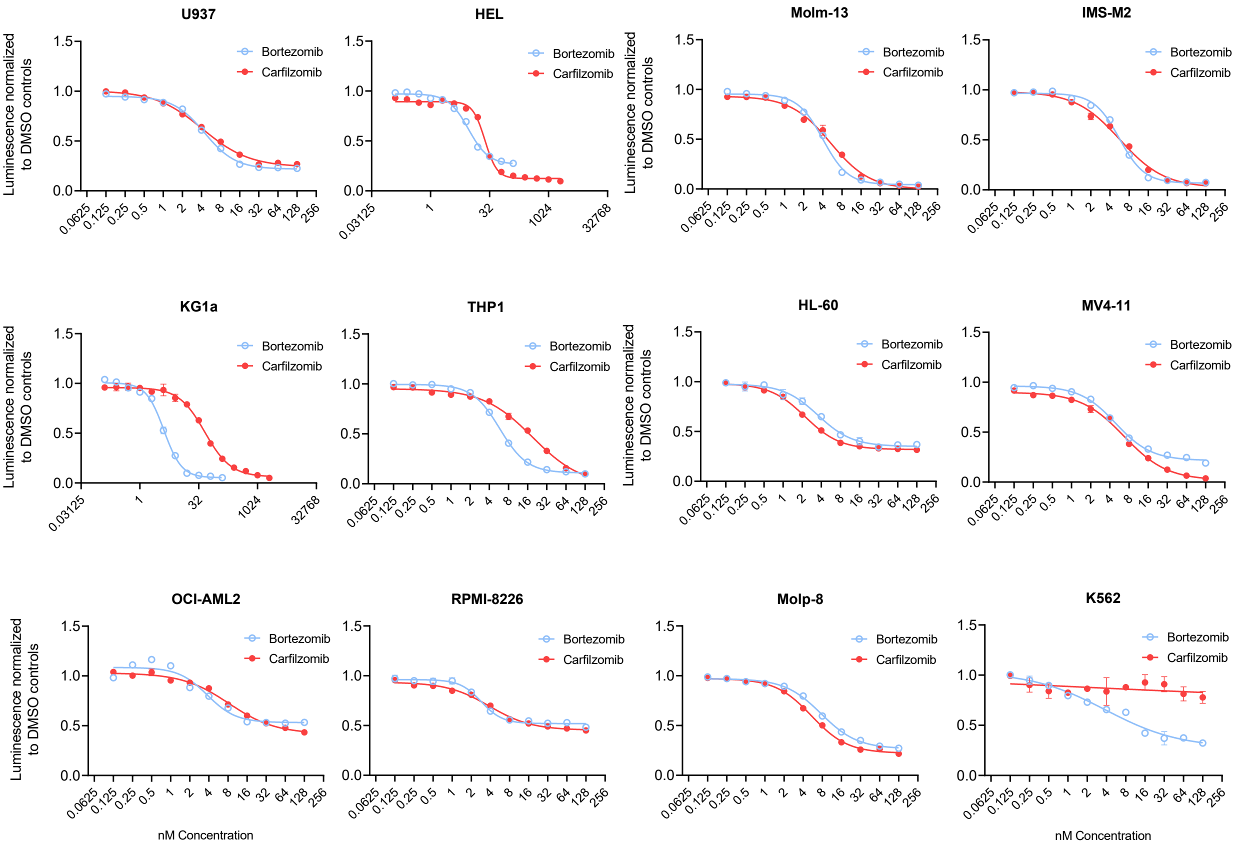

Supplementary Figure S2

Cell Line:	IC50 nM CFZ (95% CI)	IC50 nM BTZ (95% CI)
**HPSC**	301,2 (196,4 to ???)	54,97 (47,00 to 63,45)
**Resting primary NK cells**	72,39 (67,74 to 77,97)	44,57 (40,42 to 49,97)
**KG1a wt**	45,96 (40,93 to 50,25)	4,12 (3,823 to 4,455)
**HEL**	24,35 (21,53 to 26,25)	9,16 (8,252 to 10,13)
**PBMC**	22,21 (14,20 to 38,55)	30,99 (19,08 to 38,20)
**THP-1**	19,09 (16,06 to 27,61)	5,82 (5,644 to 6,003)
**HL-60 Res**	11,07 (10,13 to 12,26)	13,92 (10,91 to 18,66)
**OCI-AML2 Res**	9,47 (7,564 to 12,61)	4,63 (4,113 to 5,498)
**Activated primary NK cells**	8,29 (7,333 to 9,308)	6,61 (5,611 to 7,859)
**OCI-AML2**	8,25 (6,996 to 9,859)	4,62 (2,797 to 5,766)
**MV4-11**	6,68 (5,020 to 7,730)	4,82 (4,371 to 5,266)
**IMS-M2**	5,74 (4,979 to 6,401)	5,53 (5,139 to 5,919)
**Molp-8**	5,42 (4,892 to 5,752)	7,54 (6,801 to 8,416)
**Molm-13**	5,25 (4,255 to 5,751)	7,87 (6,979 to 8,630)
**U937**	4,13 (3,859 to 4,738)	4,30 (3,793 to 4,583)
**RPMI-8226**	3,86 (2,341 to 4,330)	2,89 (2,424 to 3,195)
**HL-60**	2,21 (1,975 to 2,343)	3,45 (2,960 to 3,960)
**Molm-13 Res**	0,77 (0,68 to 1,02)	5,76 (5,462 to 6,115)

Cell Line:	IC50 nM Cytarabine (95% CI)	IC50 nM Daunorubicin (95% CI)
**Molm-13**	401,4 (340,7 to 458,5)	70,84 (59,44 to 84,24)

Activated NK cells:	IC50 nM CFZ (95% CI)	IC50 nM BTZ (95% CI)
**aNK 1**	*6,52 (5,139 to 7,408)*	*4,88 (4,096 to 5,640)*
**aNK 2**	*5,77 (5,025 to 12,06)*	*4,87 (4,157 to 5,454)*
**aNK 3**	*5,3 (2,852 to 5,931)*	*5,05 (4,469 to 6,201)*
**aNK 4**	*6,39 (5,093 to 7,023)*	*4,46 (4,145 to 4,797)*
**aNK 5**	*10,53 (9,216 to 11,48)*	*11,03 (7,658 to 16,62)*
**aNK 6**	*12,28 (11,31 to 13,12)*	*8,19 (7,112 to 9,646)*
**aNK 7**	*11,43 (10,19 to 14,46)*	*6,61 (5,359 to 7,712)*
**aNK 8**	*8,04 (7,473 to 8,302)*	*11,67 (8,774 to 18,52)*

Resting NK cells:	IC50 nM CFZ (95% CI)	IC50 nM BTZ (95% CI)
**NK 1**	*85,07 (65,69 to 115,6)*	*44,23 (35,63 to 57,35)*
**NK 2**	*65,16 (56,64 to 75,43)*	***42,30 (36,83 to 49,76)***
**NK 3**	*80,00 (64,90 to 99,48)*	*48,66 (35,50 to 72,33)*
**NK 4**	*81,81 (64,71 to 108,2)*	*35,50 (30,67 to 43,39)*
**NK 5**	*69,34 (62,50 to 77,94)*	*46,52 (36,61 to 59,60)*
**NK 6**	*65,09 (55,50 to 76,07)*	*60,88 (50,61 to 72,57)*

Supplementary Figure S3

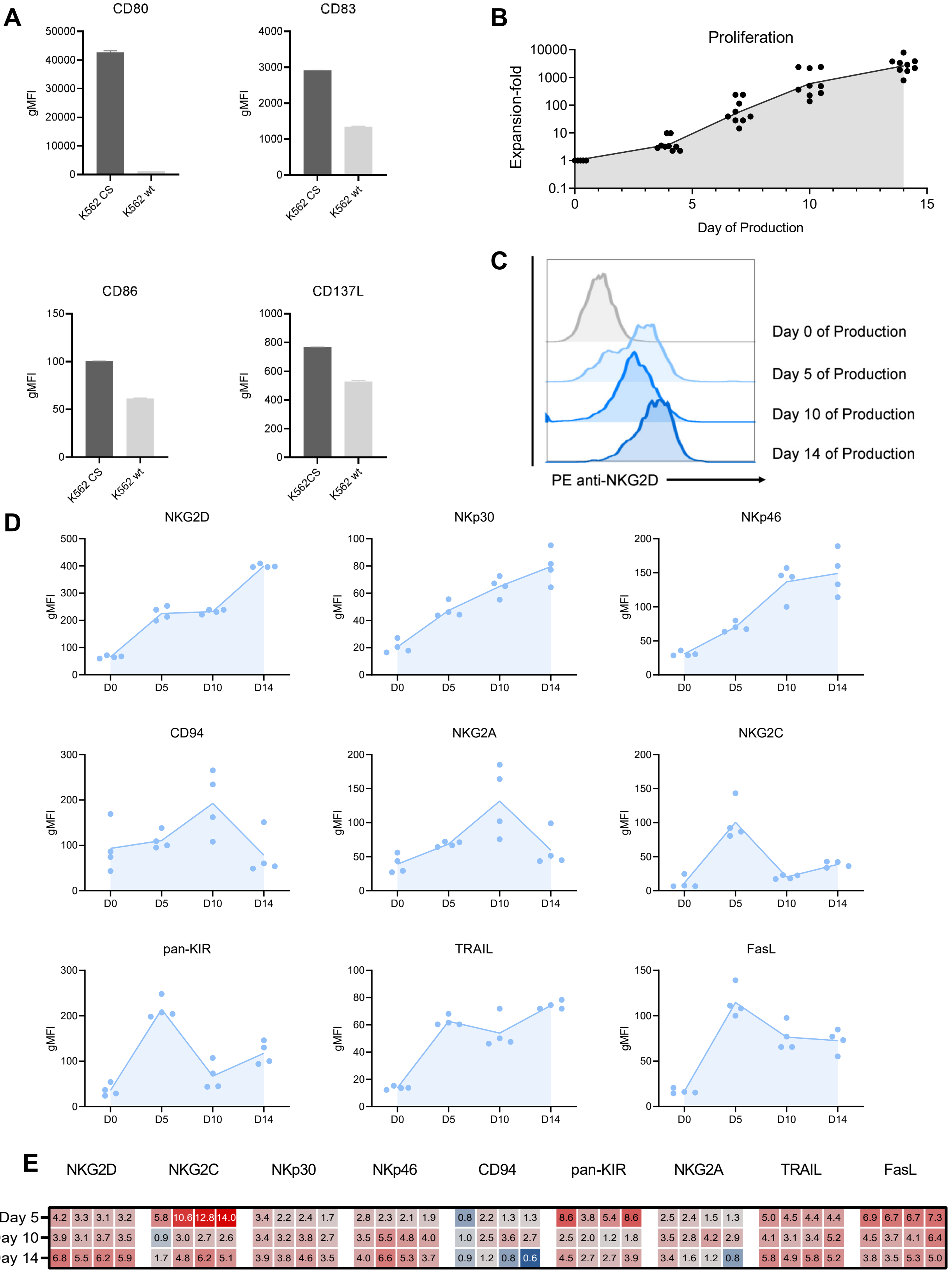

Supplementary Figure S4

Name	Clone	Dilution ratio	Company
Fc-Block TruStain FcX	-	1:100	Biolegend (422302)
7-amino-actinomycin D Viability Stain	-	1:50	Biolegend (420404)
AnnexinV BV421	-	1:100	Biolegend (640924)
APC-Conjugated Streptavidin	-	1:200	Biolegend (405207)
Biotinylated Protein L	-	1μg/mL	ThermoFisher (29997)
anti-HLA-F APC LotB302379	3D11	1:100	Biolegend (373208)
anti-HLA-ABC PE-Cy7 Lot B290872	W6/32	1:100	Biolegend (311430)
anti-HLA-E PE Lot B290135	3D12	1:100	Biolegend (342604)
anti-HLA-G AlexaFluor 488 Lot B323174	87G	1:100	Biolegend (335918)
anti-DR4 Alexa Fluor 750 Lot 1630560	69036	1:100	R&D Systems (FAB347S)
anti-DR5 PE Lot B289919	DJ-R2/4	1:100	Biolegend (307406)
anti-ULBP1 PerCP Lot ABDB0420091	170818	1:100	R&D Systems (FAB1380C)
anti-ULBP2/5/6 AlexaFluor 594 Lot 1621014	165903	1:100	R&D Systems (FAB1298T)
anti-ULBP3 Alexa Fluor 700 Lot 1630548	166510	1:100	R&D Systems (FAB1517N)
anti-TRAIL APC Lot B312412	RIK-2	1:100	Biolegend (308210)
anti-Fas BV510 Lot B317046	DX2	1:100	Biolegend (305640)
anti-FasL BV421 Lot B312573	NOK-1	1:100	Biolegend (306412)
anti-CD3 BV421	OKT3	1:100	Biolegend (317344)
anti-CD56 FITC	5.1H11	1:100	Biolegend (362546)
anti-NKG2D PE	1D11	1:100	Biolegend (320806)
anti-MICA/B PE	6D4	1:100	Biolegend (320906)

Name	Clone	Dilution ratio	Company
anti-NKp30 APC	P30-15	1:100	Biolegend (325210)
anti-NKp46 APC	9E2	1:100	Biolegend (331918)
anti-NKG2A PE-Cy7	S19004C	1:100	Biolegend (375114)
anti-NKG2C PE	S19005E	1:100	Biolegend (375004)
anti-CD80 APC	W17149D	1:100	Biolegend (375404)
anti-CD83 BV421	HB15e	1:100	Biolegend (305324)
anti-CD86 FITC	BU63	1:100	Biolegend (374204)
anti-CD33 PE-Cy7	P67.6	1:100	Biolegend (366618)
anti-CD34 APC	S20016E	1:100	Biolegend (378606)
anti-CD38 FITC	HB-7	1:100	Biolegend (356610)
anti-CD70 APC	113-16	1:100	Biolegend (355110)
anti-CD19 APC	HIB19	1:100	Biolegend (302212)
anti-CD137L PE-Cy7	5F4	1:100	Biolegend (311511)
anti-CD94 PE-Dazzle 594	DX22	1:100	Biolegend (305519)
anti-CD69 PerCP	FN50	1:100	Biolegend (310928)
anti-panKIR APC	DX27	1:100	Biolegend (312611)
Calreticulin Monoclonal Antibody	1G6A7	1μg/mL	ThermoFisher (MA5-15382)
FITC anti-mouse IgG (Rat polyclonal)	-	1:200	Biolegend (406001)
PE anti-human CD274 (PD-L1) Antibody	29E.2A3	1:100	Biolegend (329705)
APC anti-human CD273 (PD-L2) Antibody	MIH18	1:100	Biolegend (345507)

Cell lines used in each experiment:

IC50 Titration (Fig 1A-C; Fig S1-2):	Phenotype Changes (Fig 2C):	NT-NK Killing Assay (Fig 3A-B):	CAR-NK Killing Assay (Fig 4A-C):
U-937	U-937	U-937	U-937
HEL	HEL	HEL	Molm-13
Molm-13	Molm-13	Molm-13	HL-60
MV4-11	MV4-11	MV4-11	
HL-60	HL-60	HL-60	
KG1a	KG1a	KG1a	
THP-1	THP-1	THP-1	
IMS-M2	IMS-M2	IMS-M2	
OCI-AML2	K562		
RPMI8226	HL-60 Aza/Ven Res		
Molp-8	Molm-13 Aza/Ven Res		
HL-60 Aza/Ven Res			
Molm-13 Aza/Ven Res			
OCI-AML2 Aza/Ven Res			

Blocking antibodies and death ligands:

Name:	Biological source	Dilution ratio	Company
rhTRAIL	-	Final concentration of 100ng/ml	Biolegend (752904)
rhFasL	-	Final concentration of 100ng/ml	Biolegend (589402)
anti-NKG2D Blocking Antibody	Mouse, monoclonal, 149810	Final concentration of 10μg/mL	R&D Systems (MAB139-SP)
Purified Mouse IgG1, κ Isotype Ctrl Antibody	Mouse, monoclonal, MOPC21	Final concentration of 10μg/mL	Biolegend (400101)
anti-HLA ABC	Mouse, monoclonal, W6/32	Final concentration of 10μg/mL	Biolegend (311402)
Purified anti-human CD178 (Fas-L) Antibody	Mouse, monoclonal, NOK-1	Final concentration of 10μg/mL	Biolegend (306402)
Purified anti-human CD253 (TRAIL) Antibody	Mouse, monoclonal, RIK-2	Final concentration of 10μg/mL	Biolegend (308202)

Western Blot antibodies:

Name and MW (kDa)	Biological source	Dilution ratio	Company
Anti-rabbit IgG, HRP-linked Antibody	Mouse, monoclonal	1:10000	Cell Signaling Technology (7074P2)
Anti-NFkB p65 antibody (65)	Rabbit, polyclonal	1:1000	ThermoFisher (51-0500)
Phospho-NF-κB p65 Ser536 (65)	Rabbit, monoclonal	1:1000	Cell Signaling Technology (3033T)
Anti-β-Actin-HRP (45)	Mouse, monoclonal	1:1000	Cell Signaling Technology (12262S)
Name and MW (kDa)	Biological source	Dilution ratio	Company

qPCR Primers:

Target:	Fwd:	Rev:
ULBP-1	TGCAGGCCAGGATGTCTTGT	CATCCCTGTTCTTCTCCCACTTC
ULBP-2	CAGAGCAACTGCGTGACATT	GGCCACAACCTTGTCATTCT
ULBP-3	GGATTTCACACCCAGTGGAC	GCCTCTTCTTCCTGTGCATC
MICA/B	ACAATGCCCCAGTCCTCCAGA	ATTTTAGATATCGCCGTAGTTCCT

Supplementary Figure S5

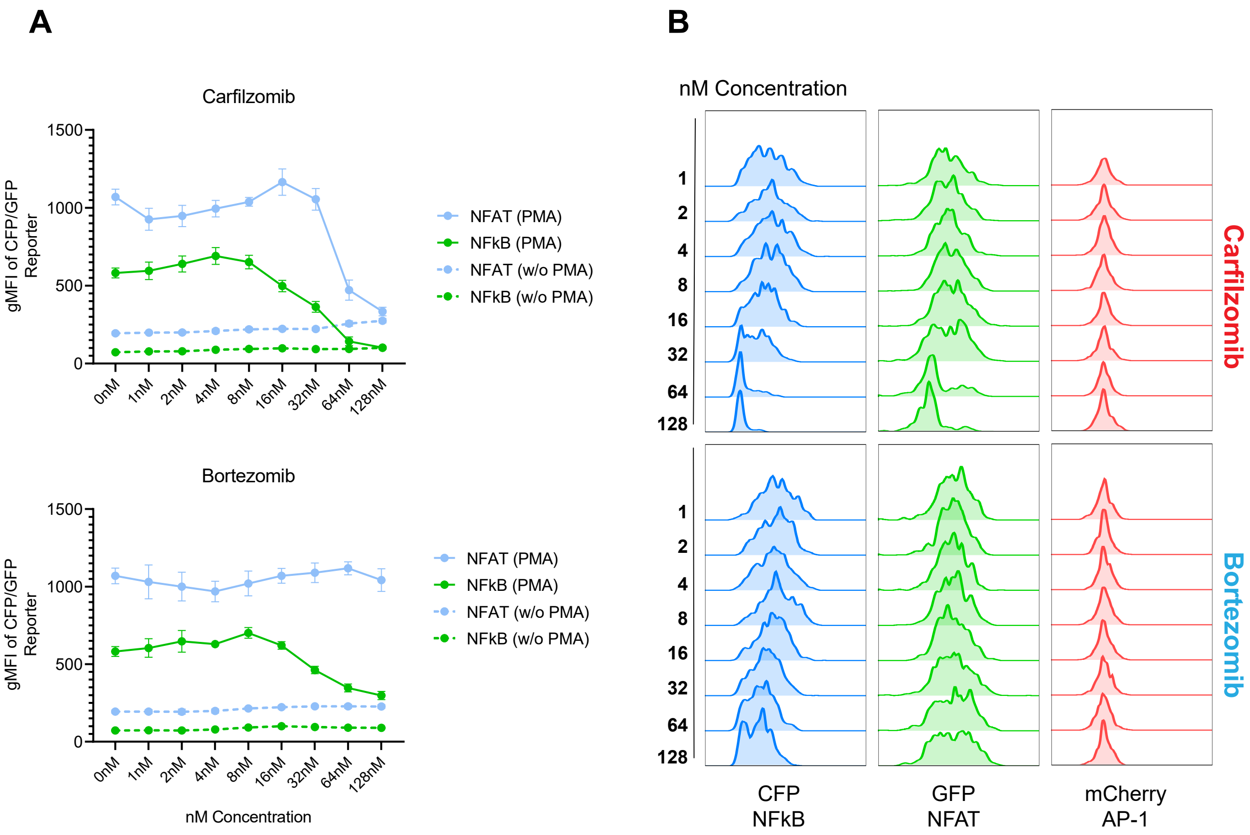

Supplementary Figure S6

A

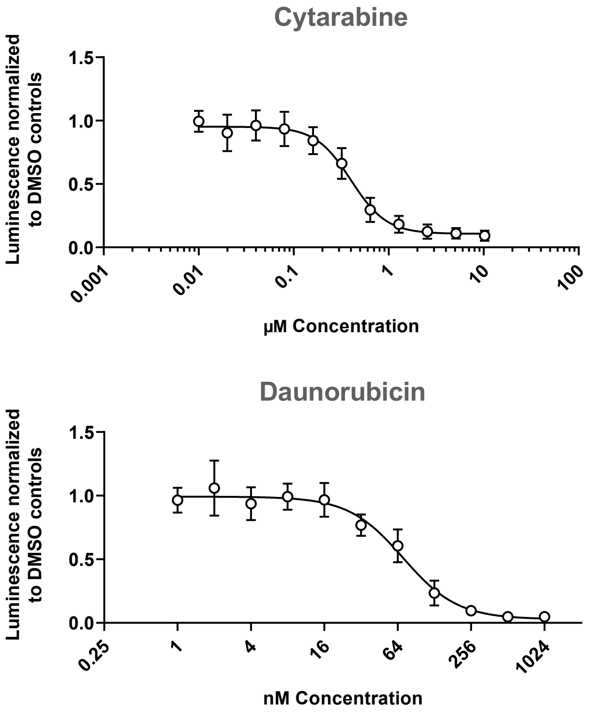

B

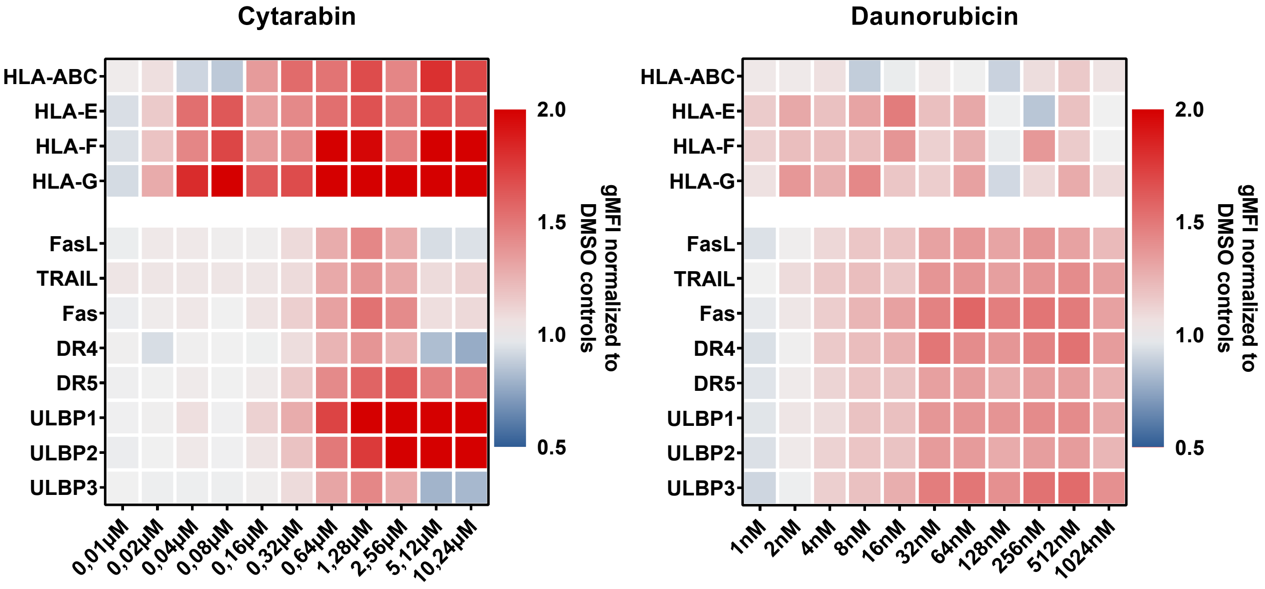

Supplementary Figure S7

A

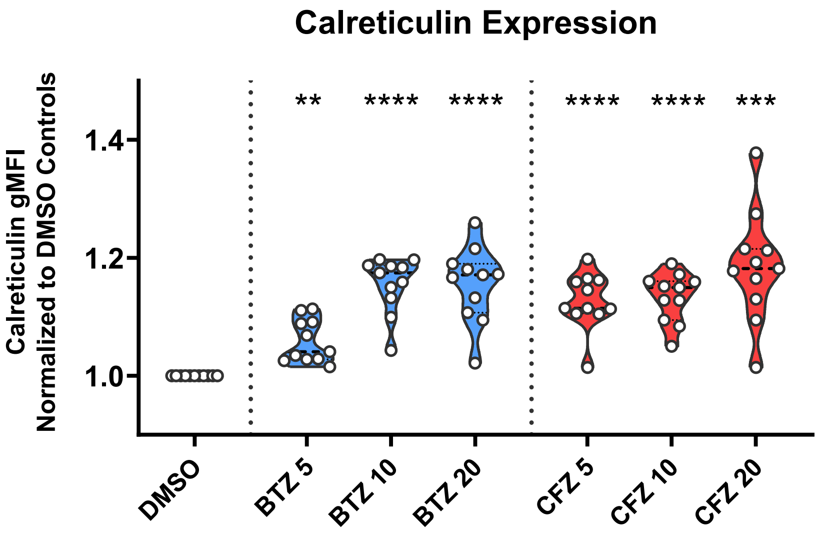

B

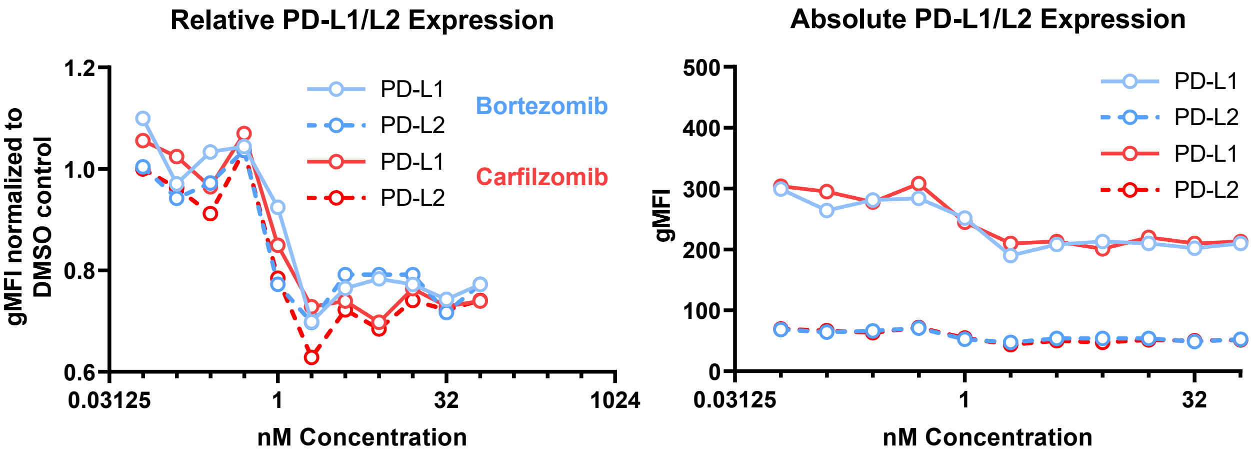

Supplementary Figure S8

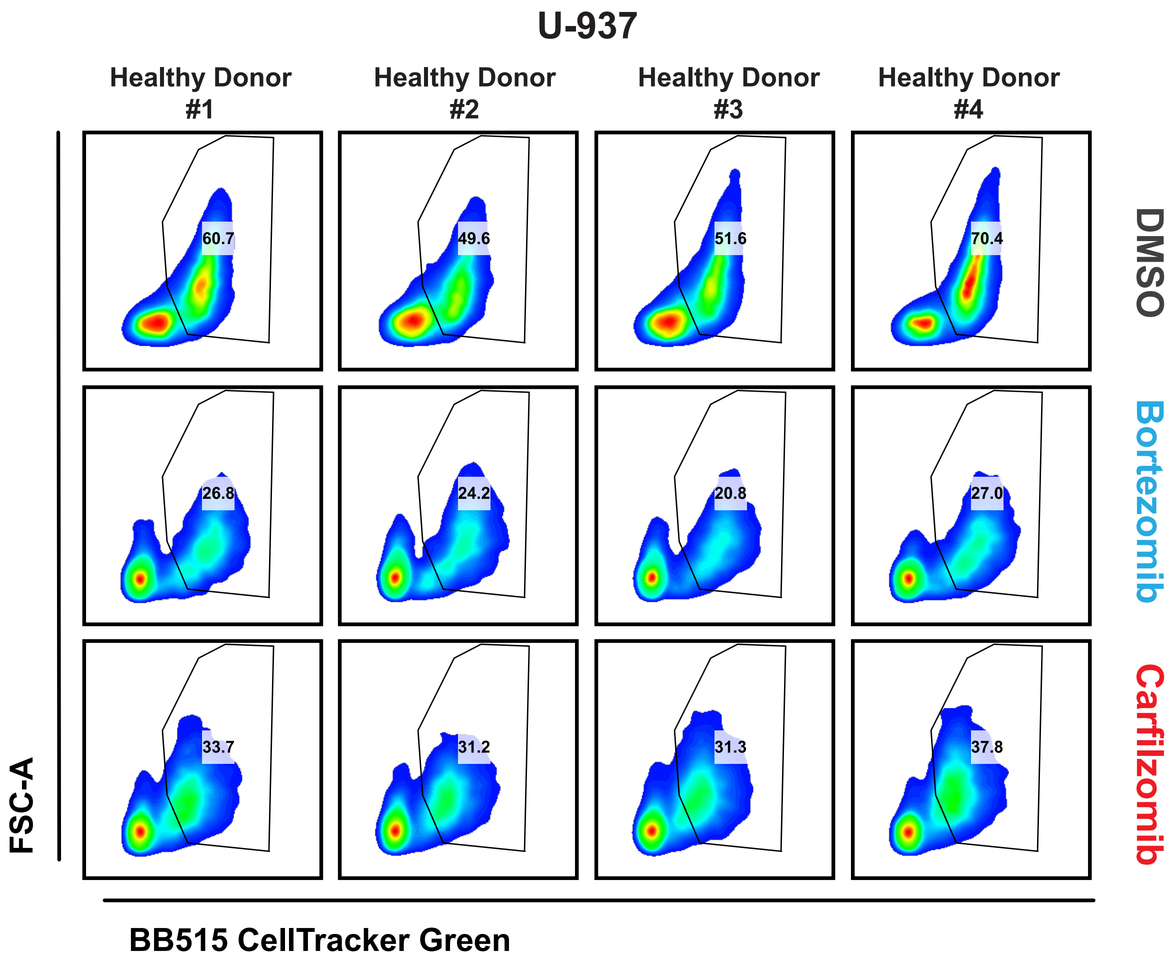

Supplementary Figure S9

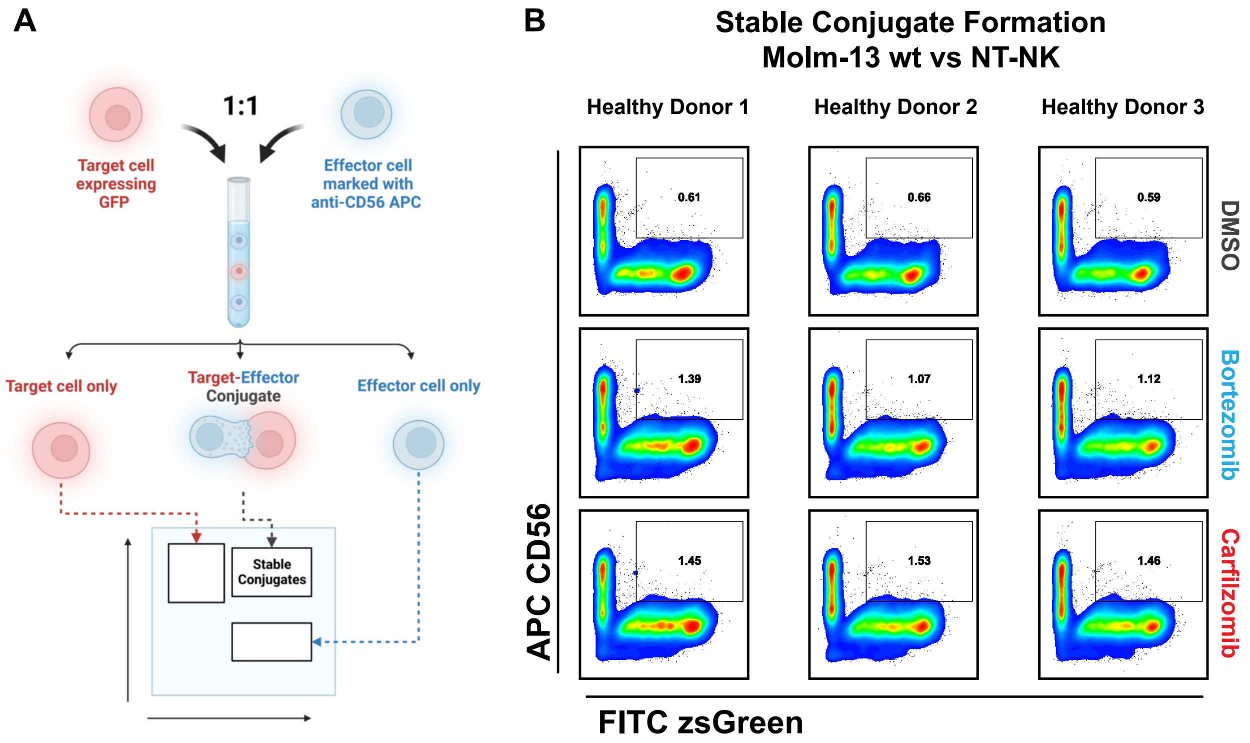

Supplementary Figure S10

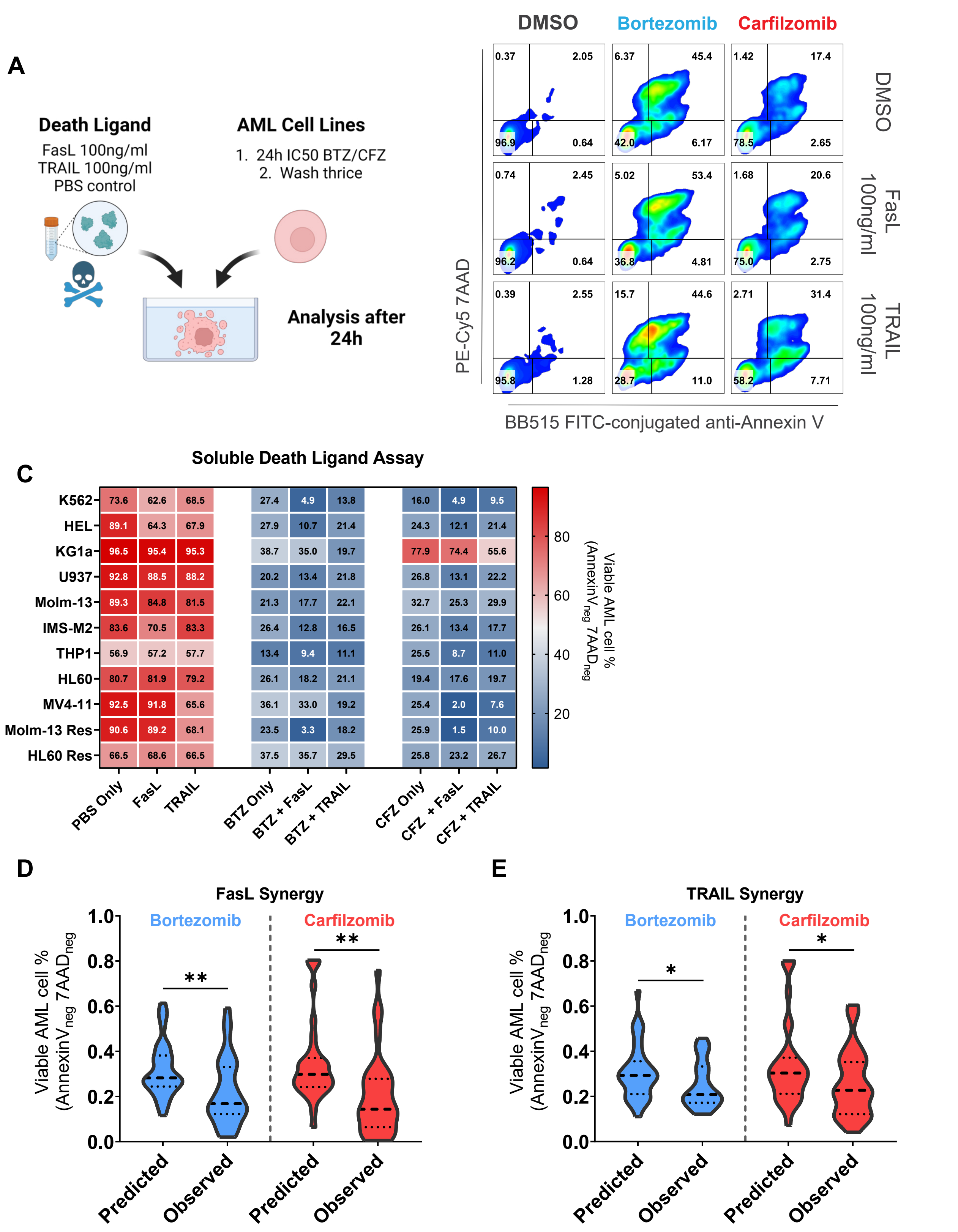

Supplementary Figure S11

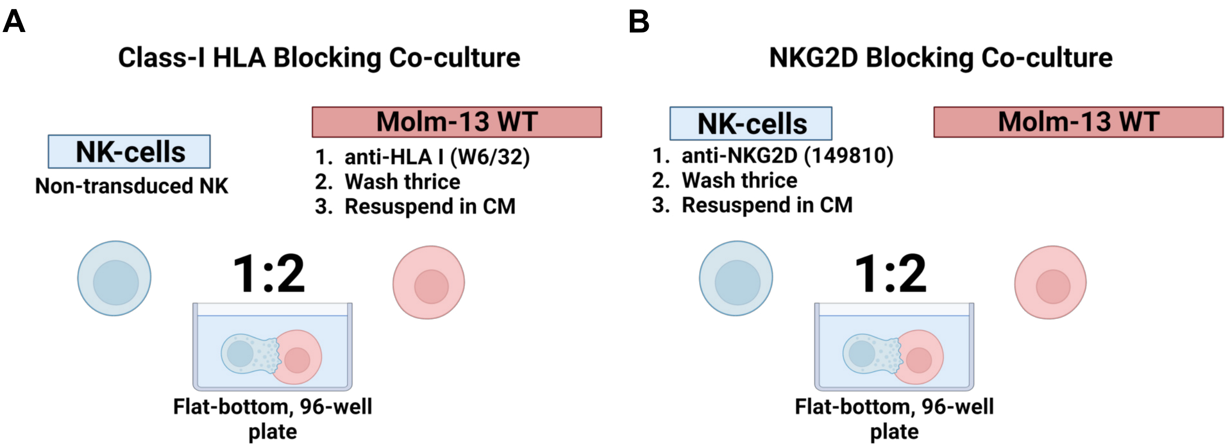

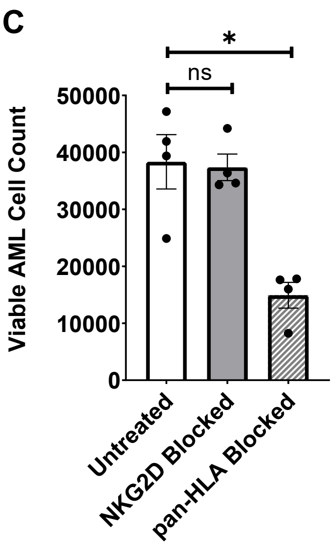

D

Measured Parameters - gMFI without Proteasomal Inhibition

	K562	HEL	KG-1a	U-937	Molm-13	IMS-M2	THP-1	HL-60	MV4-11	HL-60 Res	Molm-13 Res
HLA-ABC	103.0	224.0	823.0	767.0	521.0	914.0	66.8	82.2	496.0	875.0	234.0
HLA-E	73.2	62.9	78.4	70.6	56.5	77.1	111.0	65.5	99.0	66.8	78.4
HLA-F	48.8	39.8	47.5	38.5	34.7	44.9	57.8	48.8	69.4	44.9	59.1
HLA-G	92.5	83.5	44.9	66.8	52.6	55.2	318.0	82.2	129.0	55.2	69.4
FasL	79.6	64.2	28.2	29.5	27.0	28.2	44.9	39.8	33.4	59.1	34.7
TRAIL	37.2	29.5	19.3	14.1	14.1	19.3	20.5	19.3	20.5	30.8	18.0
Fas	351.0	285.0	121.0	142.0	147.0	139.0	263.0	192.0	142.0	178.0	170.0
DR4	241.0	168.0	222.0	78.4	82.2	131.0	136.0	129.0	211.0	194.0	116.0
DR5	274.0	97.7	56.5	43.6	55.2	104.0	173.0	111.0	99.0	148.0	152.0
ULBP1	347.0	181.0	86.1	104.0	113.0	148.0	242.0	171.0	185.0	209.0	233.0
ULBP2	306.0	122.0	64.2	51.4	66.8	116.0	192.0	130.0	112.0	173.0	171.0
ULBP3	77.1	55.2	75.8	28.2	28.2	46.2	38.5	32.1	66.8	69.4	32.1
MICA/B	56.5	55.2	321.0	20.5	34.7	314.0	199.0	52.6	277.0	20.5	860.0

Supplementary Figure S12

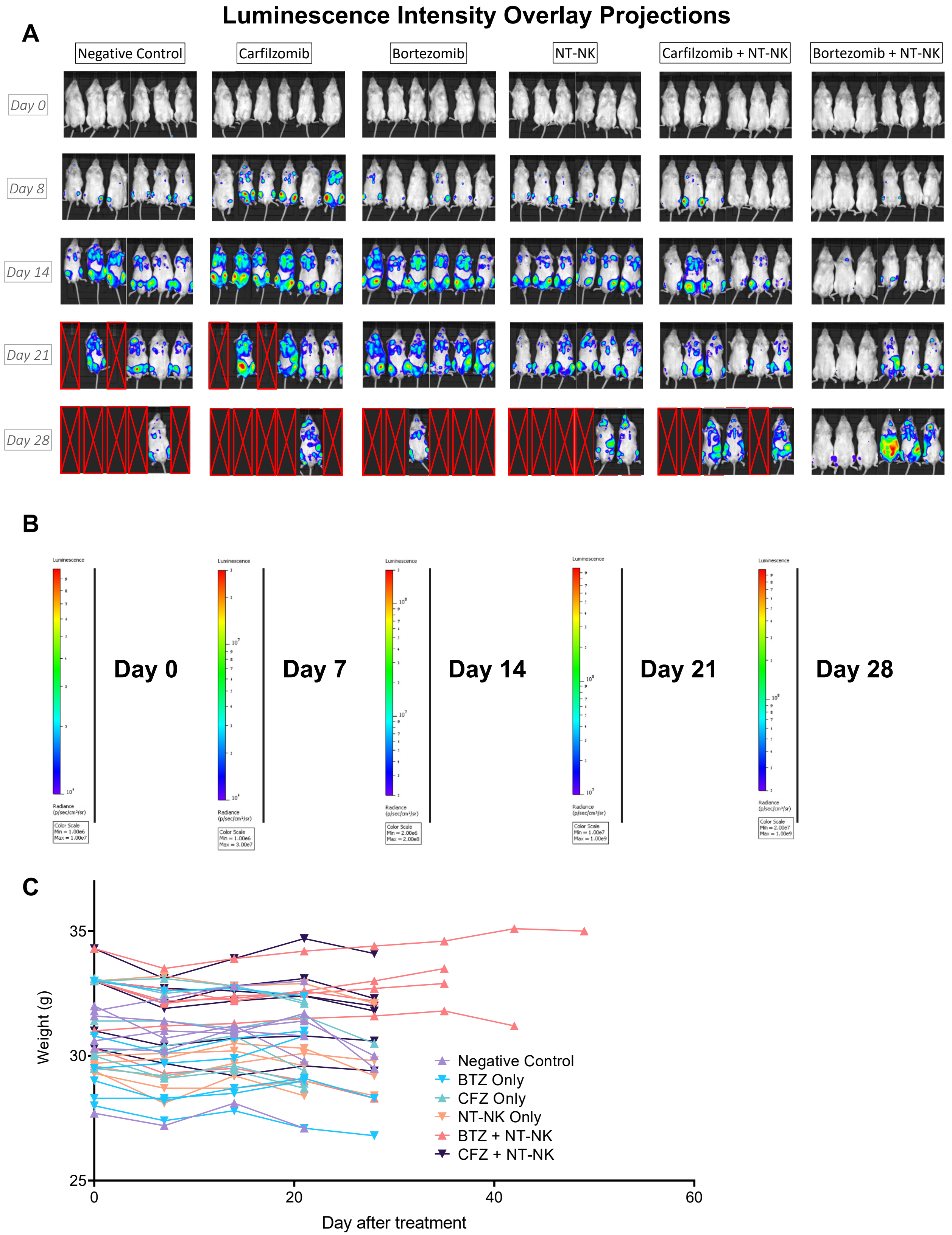

Supplementary Figure S13

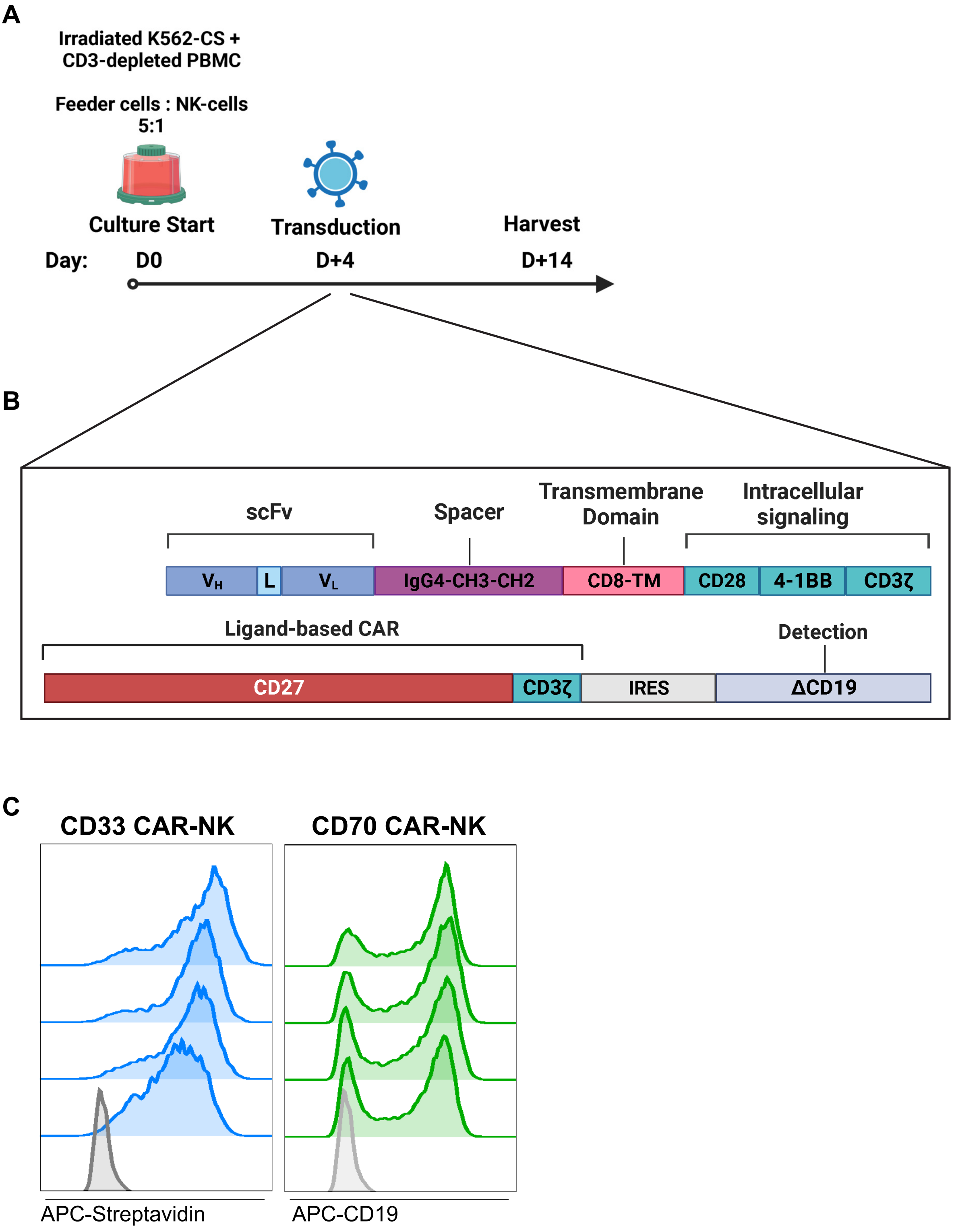

Supplementary Figure S14

A

Basic Information - Primary AML Samples

Population:	AML#1	AML#2	AML#3	AML#4	AML#5	AML#6
LSC% (CD34+ CD38-)	0.0	26,5	0,4	83,1	0,8	0,1
CD33%+	79,2	95	86,5	98,6	78,1	77,8
CD70%+	98,6	78	99,9	99,8	99,1	99,2
**gMFI:**	**AML#1**	**AML#2**	**AML#3**	**AML#4**	**AML#5**	**AML#6**
CD33 gMFI	225	859	262	902	231	203
CD70 gMFI	1469	259	5498	1076	4796	2600

B

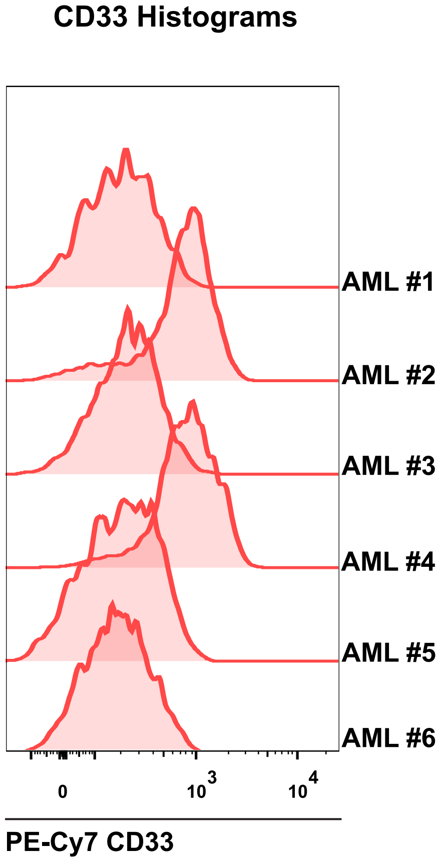

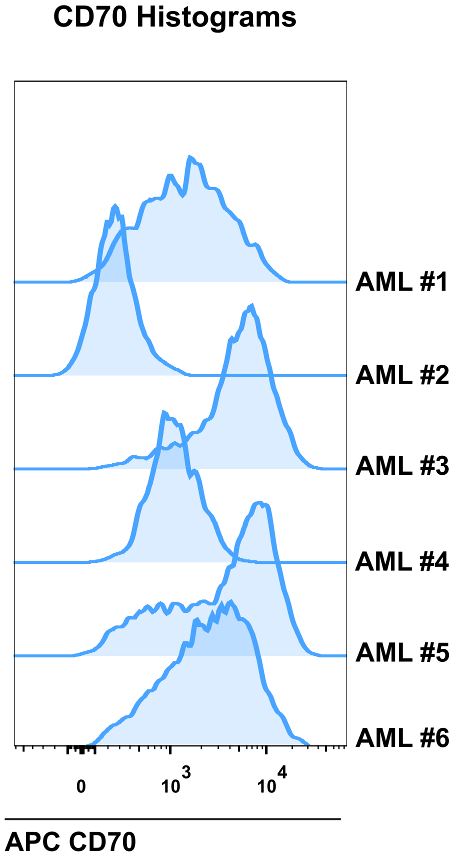

C

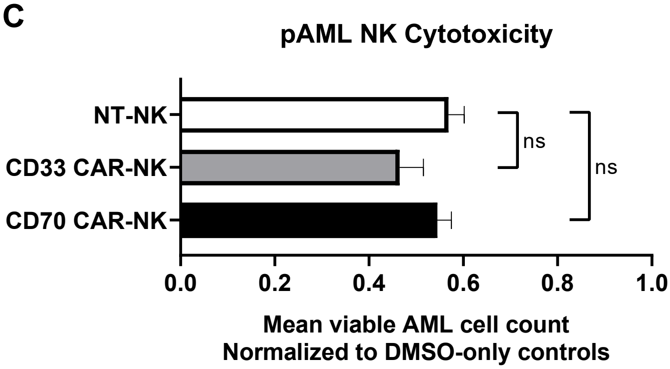

Supplementary Figure S15

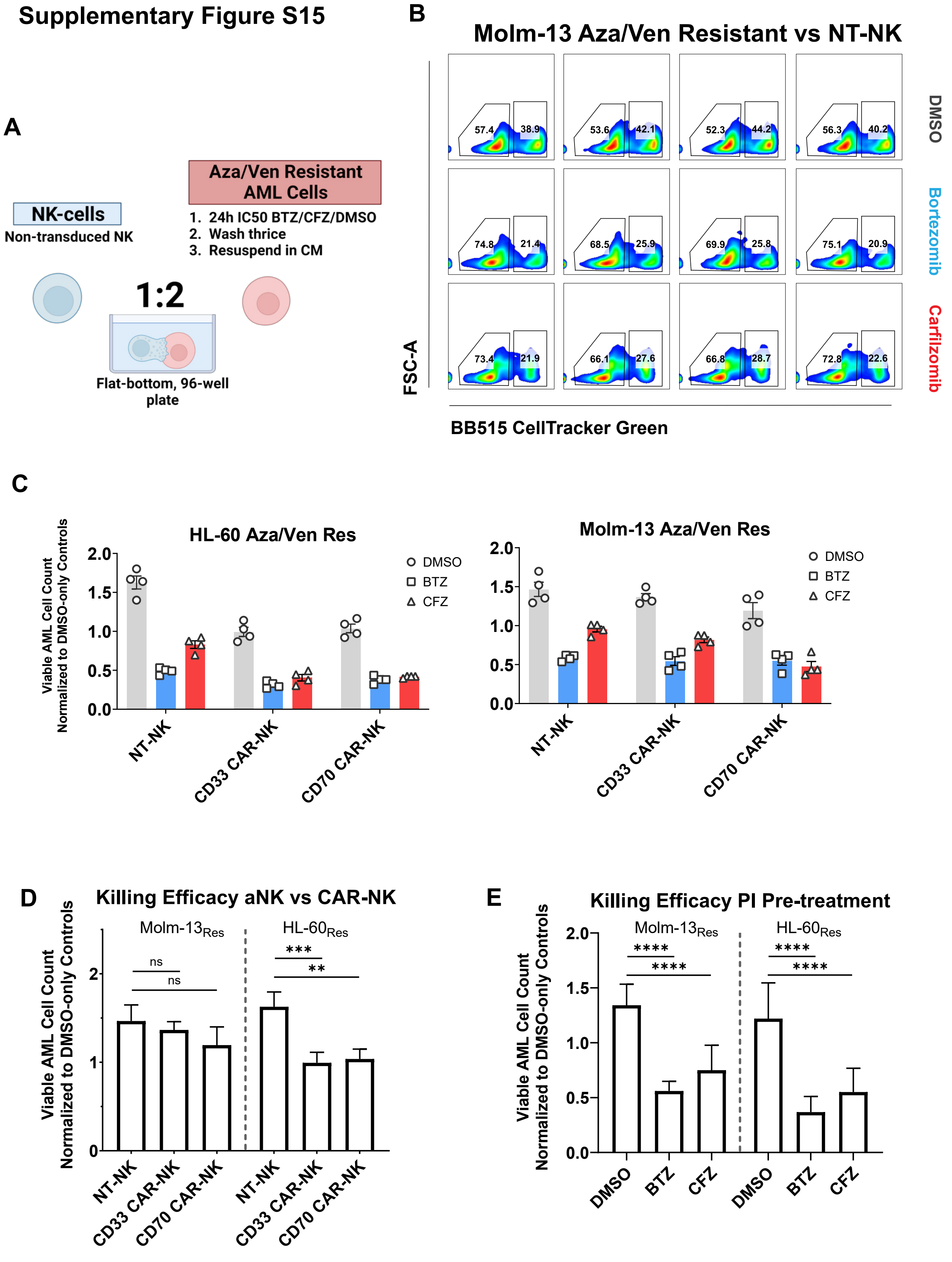

Supplementary Figure S16

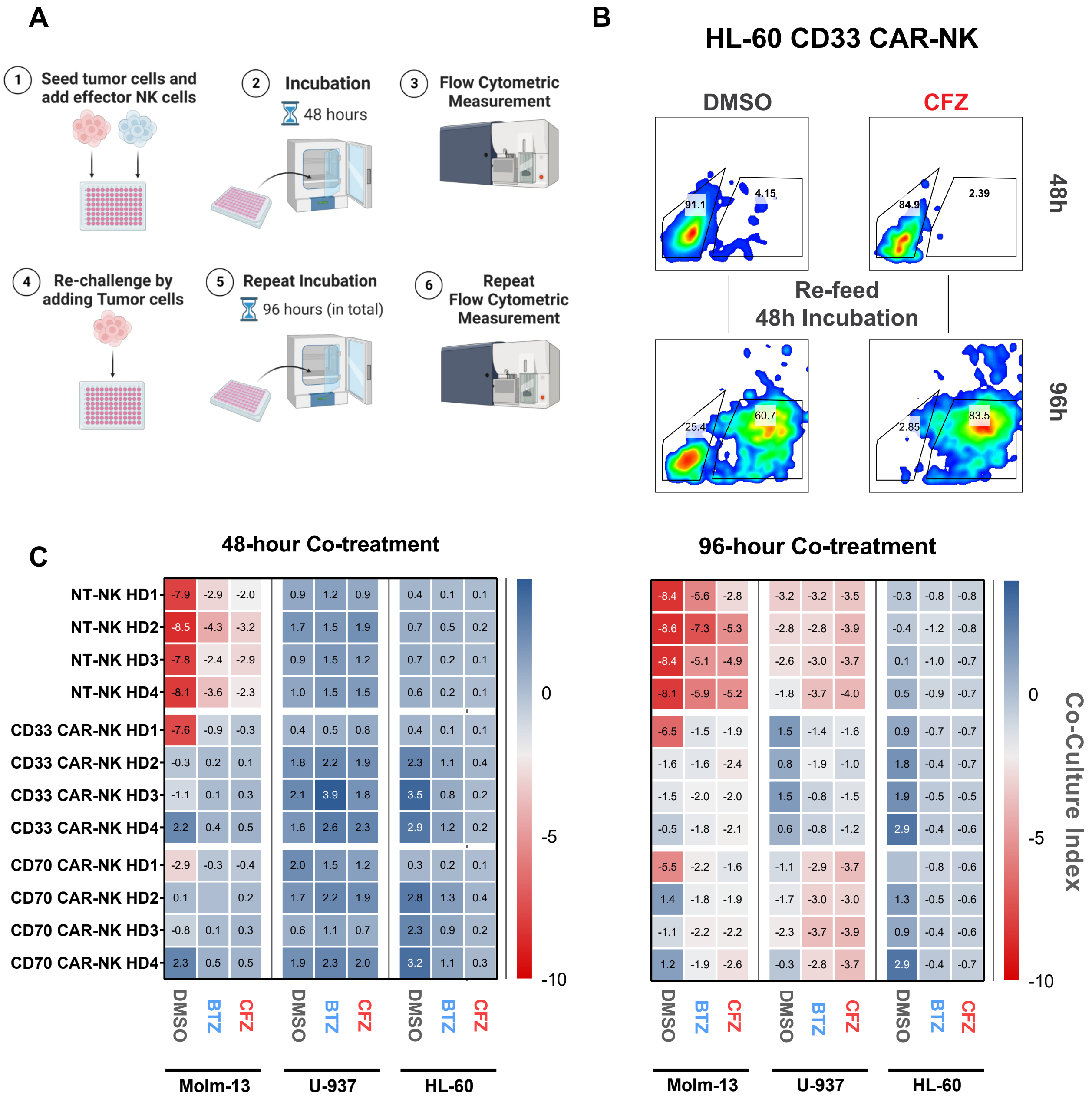

Co-Culture Index =
Fold-change Effector - Fold-change tumor
Low Co-Culture Index --> poor tumor control

Supplementary Figure S17

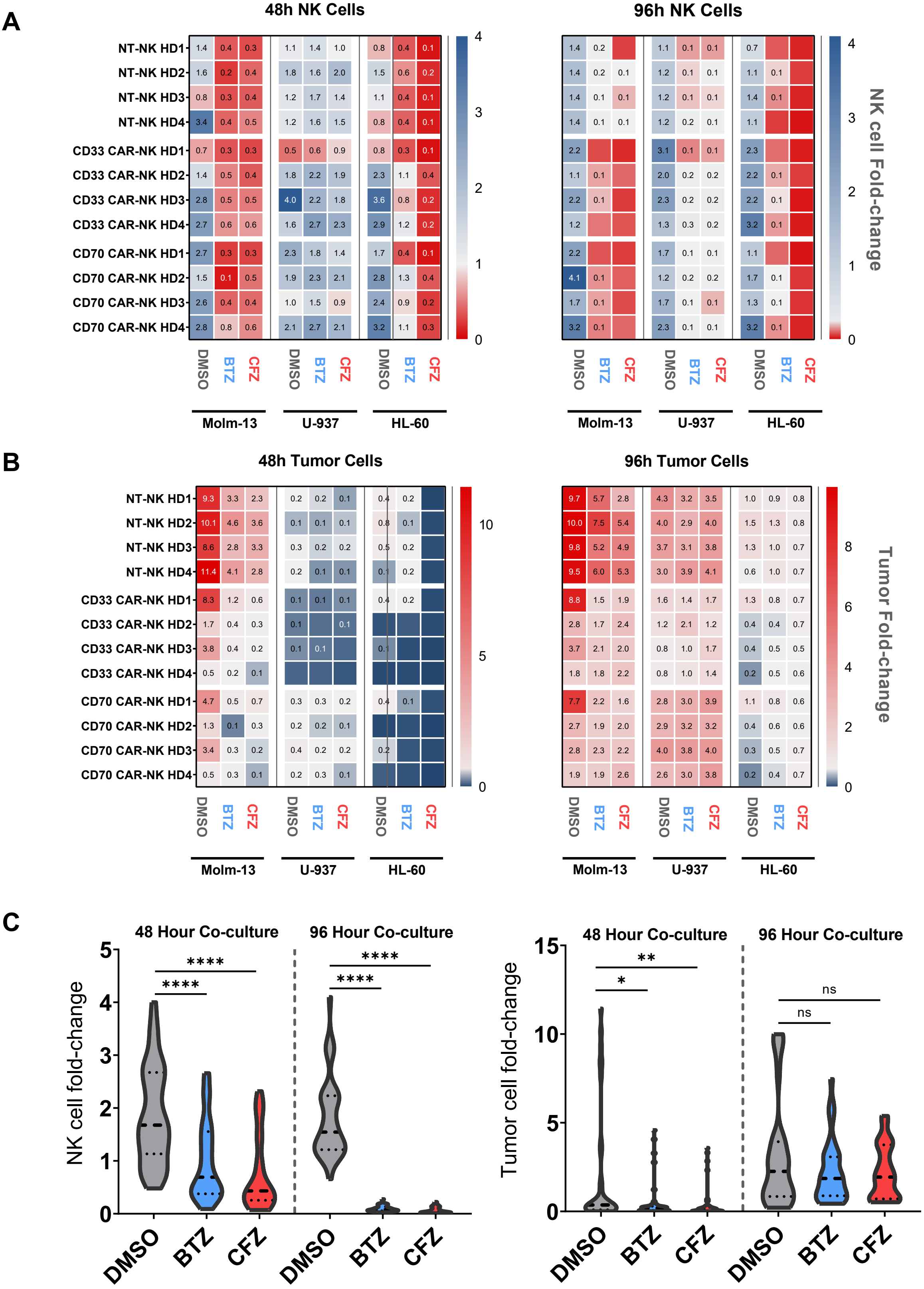

Supplementary Figure S18

A

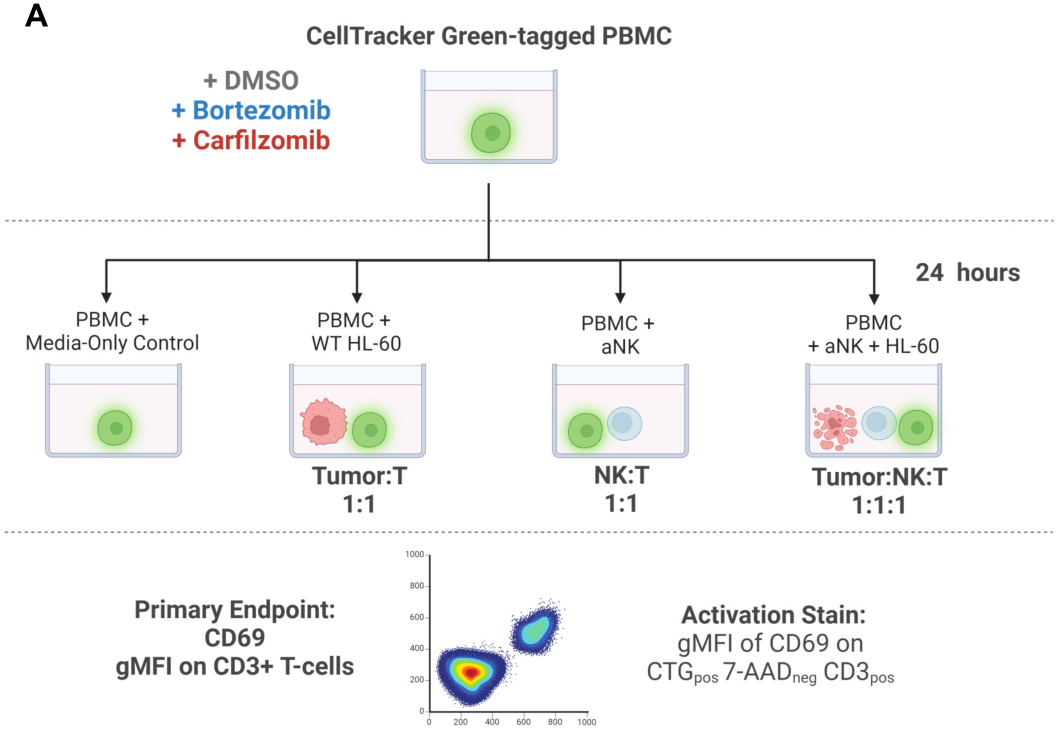

B

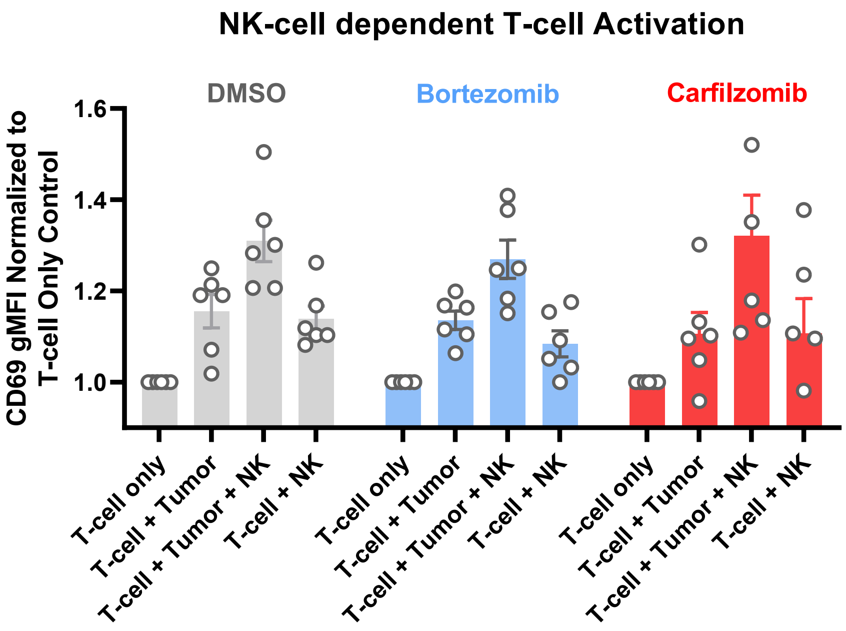

Supplementary Figure S19

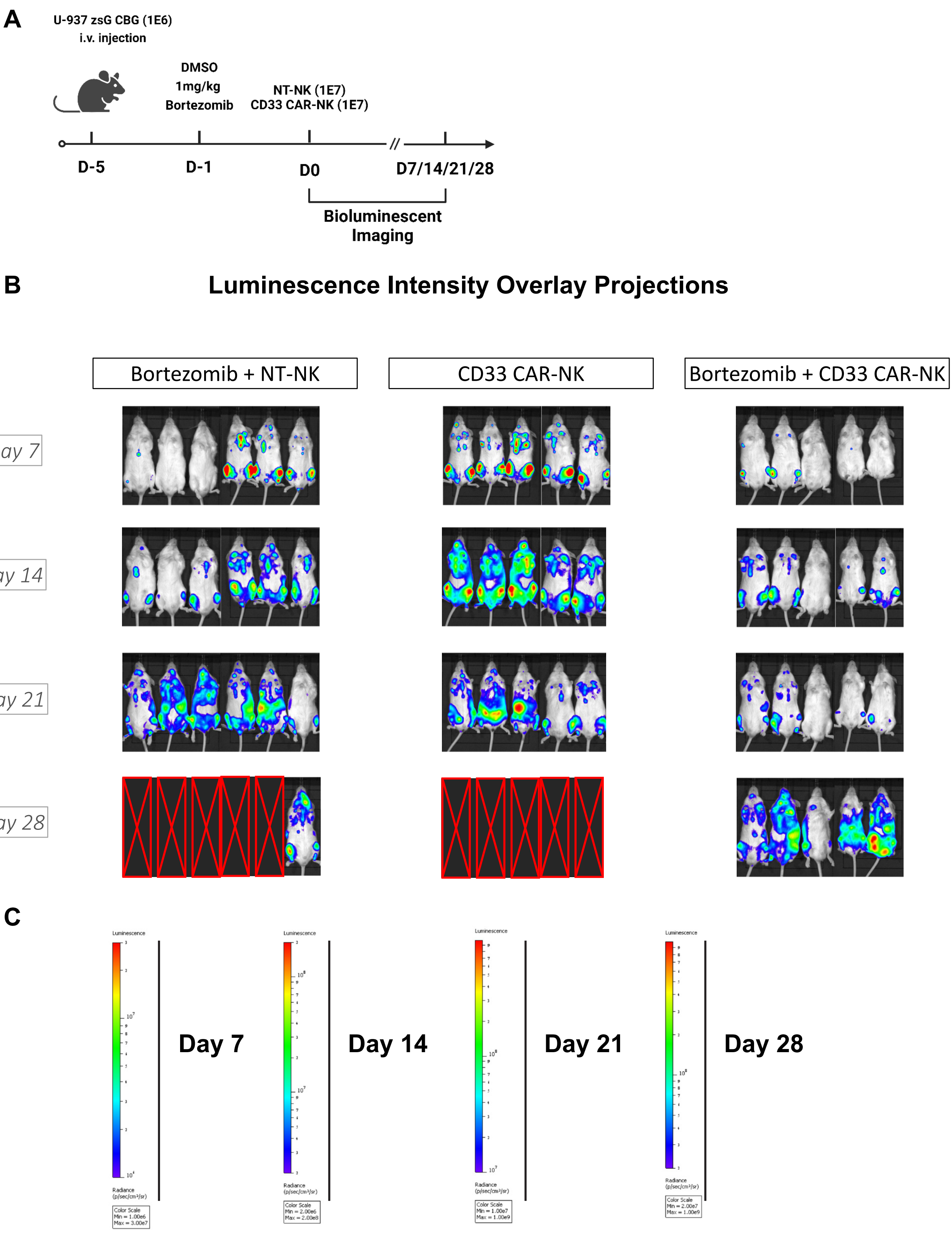

Supplementary Figure S20

A

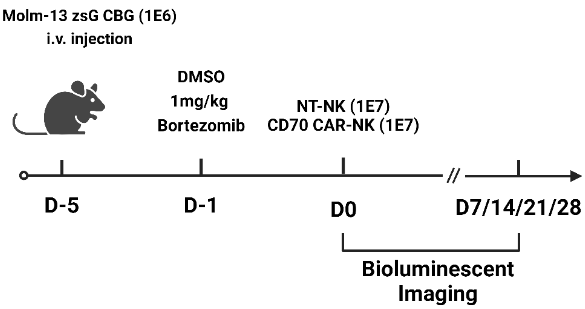

B

Luminescence Intensity Overlay Projections

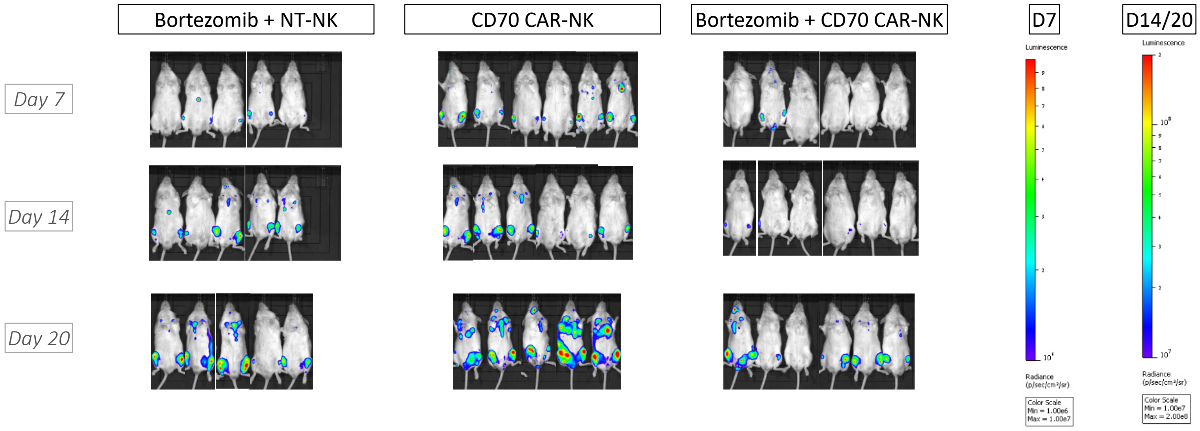

Supplementary Figure S21

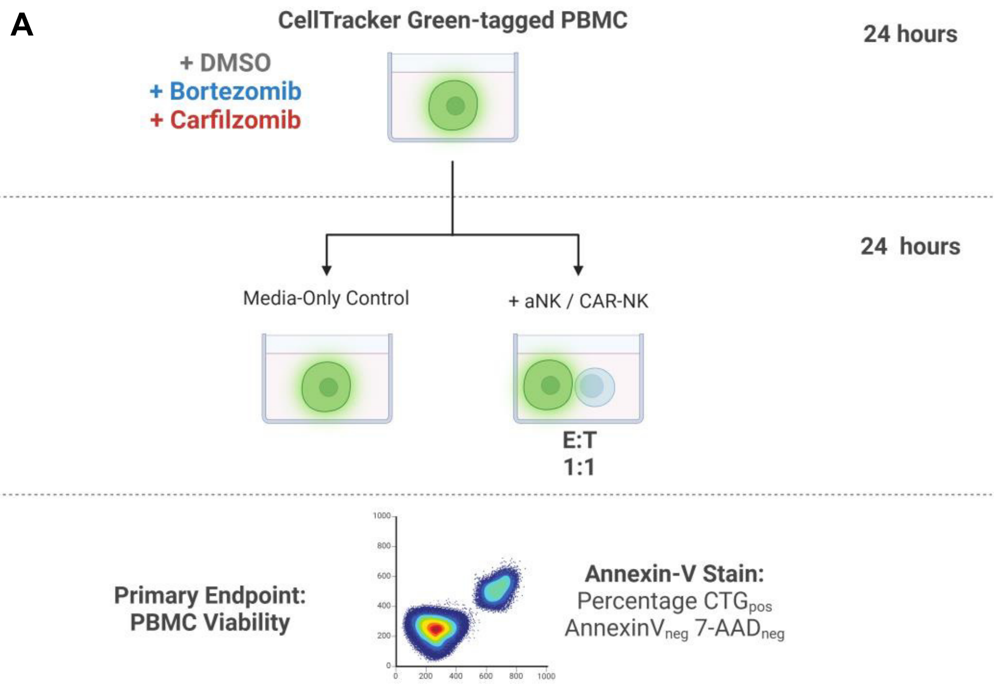

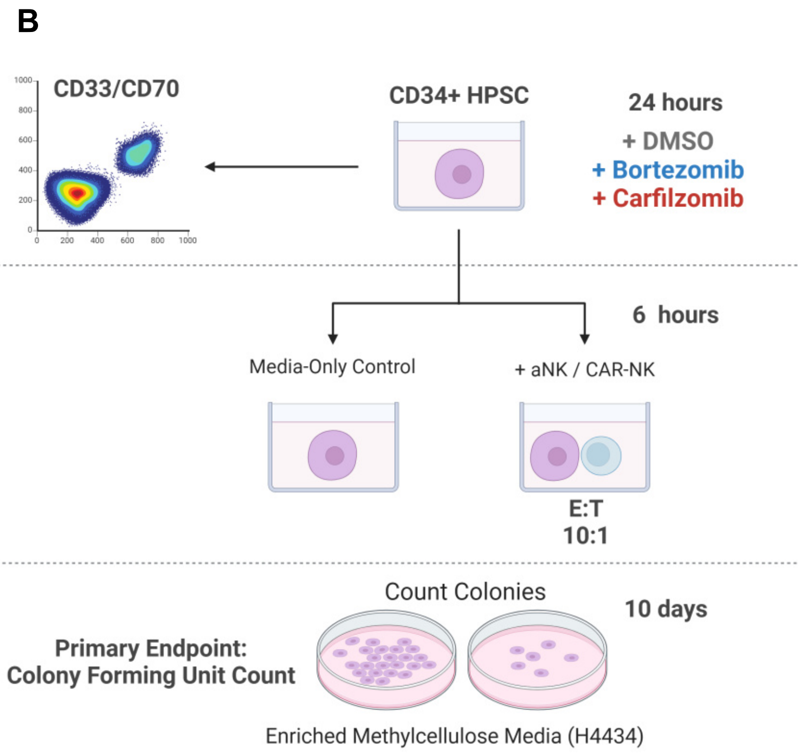

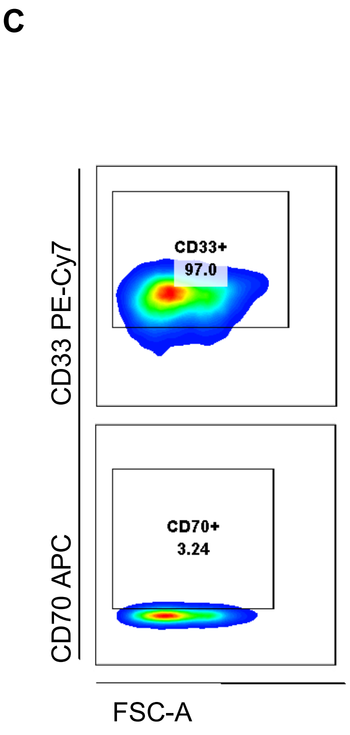

Supplementary Figure S22

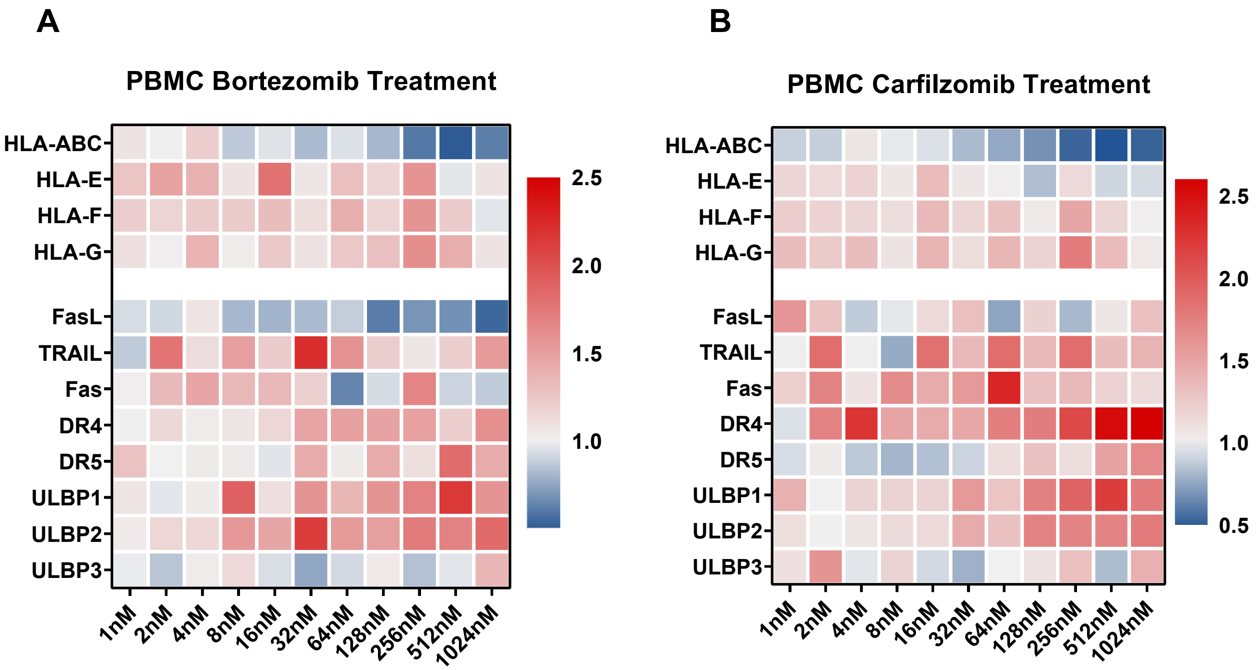

Supplement: Supplementary file 1 — 1. [file 13045_2024_1604_MOESM1_ESM.pdf]
